# *Hypnea musciformis* Seaweed Extract Protected Human Mesenchymal Stem Cells From Oxidative Stress Through NRF2 Activation

**DOI:** 10.1002/fsn3.4615

**Published:** 2024-11-27

**Authors:** Andreas Goutas, Nikolaos Goutzourelas, Alkistis Kevrekidou, Dimitrios Phaedon Kevrekidis, Paraskevi Malea, Christina Virgiliou, Andreana N. Assimopoulou, Varvara Trachana, Nikolaos Kollatos, Tafa Moustafa, Ming Liu, Xiukun Lin, Dimitrios Komiotis, Dimitrios Stagos

**Affiliations:** ^1^ Department of Biochemistry and Biotechnology, School of Health Sciences University of Thessaly Larissa Greece; ^2^ Department of Biology, Faculty of Medicine University of Thessaly Larissa Greece; ^3^ Laboratory of Organic Chemistry, School of Chemical Engineering Aristotle University of Thessaloniki Thessaloniki Greece; ^4^ Environmental Engineering Laboratory, Department of Chemical Engineering Aristotle University of Thessaloniki Thessaloniki Greece; ^5^ Laboratory of Forensic Medicine and Toxicology, Department of Medicine Aristotle University of Thessaloniki Thessaloniki Greece; ^6^ Department of Botany, School of Biology Aristotle University of Thessaloniki Thessaloniki Greece; ^7^ Laboratory of Analytical Chemistry, School of Chemical Engineering Aristotle University of Thessaloniki Thessaloniki Greece; ^8^ Key Laboratory of Marine Drugs, Ministry of Education, School of Medicine and Pharmacy Ocean University of China Qingdao China; ^9^ Laboratory for Marine Drugs and Bioproducts of Qingdao National Laboratory for Marine Science and Technology Qingdao China; ^10^ Department of Pharmacology, School of Pharmacy Southwest Medical University Luzhou China

**Keywords:** antioxidant, *Hypnea musciformis*, macroalgae, mesenchymal cells, NRF2, seaweeds

## Abstract

Previous studies have shown that *Hypnea musciformis* seaweed extracts (HMEs) possess antioxidant properties, but the molecular mechanisms accounting for this activity are not known. Thus, the present study investigated the molecular mechanisms through which HME exerted its antioxidant activity in human mesenchymal stem cells (WJ‐MSCs). After the isolation of HME, its chemical composition was analyzed with gas chromatography mass spectrometry, indicating that it contained amino acids, organic acids, organic amides, sugar alcohols, saturated fatty acids, hydrogenated diterpene alcohols, and other organic compounds. Afterward, HME was shown in vitro to scavenge DPPH^·^, ABTS^·+^, ^·^OH, and O_2_^·−^ radicals, possess reducing activity, and protect from ROO^·^‐induced DNA strand breakage. Finally, the results showed that HME treatment of WJ‐MSCs prevented H_2_O_2_‐induced oxidative stress by decreasing lipid peroxidation, protein oxidation, reactive oxygen species levels, and DNA damage and by increasing glutathione levels. Moreover, our findings showed for the first time that HME's antioxidant activity in WJ‐MSCs was mediated through the activation of NRF2, which upregulated the expression of the antioxidant proteins GCLC, GSR, HMOX1, SOD1, TXN, and GPX1. These results provide new insights into *H. musciformis* ' antioxidant properties, which could help substantially its use as a food supplement or for developing biofunctional foods.

## Graphical abstract

Extract of *Hypnea musciformis* seaweed collected from the Mediterranean Sea contained polyphenols, flavonoids, proteins, amino acids, organic acids, organic amides, sugar alcohols, saturated fatty acids, fatty acid esters, hydrogenated diterpene alcohols, and other organic compounds. HME treatment of human mesenchymal stem cells (WJ‐MSCs) prevented H_2_O_2_‐induced oxidative stress by decreasing lipid peroxidation, protein oxidation, ROS levels, and DNA damage and by increasing glutathione. HME's antioxidant activity was mediated through activation of NRF2, which upregulated the expression of the antioxidant proteins GCLC, GSR, HMOX1, SOD1, TXN, and GPX1. These results give new insight for *Hypnea musciformis* antioxidant properties and its possible use as food supplement or for developing biofunctional foods preventing from oxidative stress‐induced pathologies.

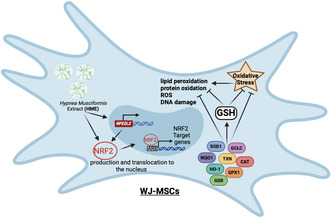

## 1 Introduction

Marine macroalgae, also known as seaweeds, play a vital role in the structure and functioning of coastal and estuarine environments (Gubelit 2022). There are over 1200 species of macroalgae, which are frequently discovered in both freshwater and marine environments. Furthermore, seaweeds play a crucial role as primary producers in marine ecosystems, contributing to over 40% of the global oxygen supply (Healy et al. 2023). Recently, there has been great interest in researching seaweeds as a valuable source of human nutrition since they contain compounds with nutritional value (e.g., vitamins such as riboflavin, niacin and folic acid, as well as minerals and trace elements) (Healy et al. 2023; Aakre et al. 2023; Samani et al. 2023). They also possess various components, such as proteins, polysaccharides, and phenolic compounds, which exhibit important bioactivities like anticancer, antimicrobial, antiviral, anti‐inflammatory, anti‐obesity, and antioxidant properties (Healy et al. 2023; Samani et al. 2023).

*Hypnea musciformis* (Wulfen) J.V. Lamouroux is a red marine macroalga found worldwide and holds significance for its bioactive compounds, especially for sulfated polysaccharides (e.g., carrageenans), which have beneficial properties for human health, such as anticancer, anti‐inflammatory, and antioxidant effects (Balamurugan et al. 2013; de Brito et al. 2013; Souza et al. 2018; Rozo et al. 2019). Regarding the antioxidant activity, *H. musciformis* extracts (HMEs) were demonstrated in vitro to scavenge free radicals (Rozo et al. 2019; Hmani et al. 2021), inhibit lipid peroxidation (Rozo et al. 2019), and protect human low‐density lipoproteins (LDLs) from oxidation, a major causative factor of heart diseases (Rozo et al. 2019). Vatan, Celikler, and Yildiz (2011) demonstrated that HME inhibited reactive oxygen species (ROS)‐induced chromosomal damage in human lymphocytes. Moreover, Balamurugan et al. (2017) showed that HME administration decreased lipid peroxidation in rat plasma, erythrocytes, and liver and mammary tissues. In addition, after extract administration of HME to rats, the activity of antioxidant enzymes such as superoxide dismutase (SOD), catalase (CAT), and glutathione peroxidase (GPX) and the levels of antioxidant molecule glutathione (GSH) were increased in plasma, erythrocytes, and mammary tissue (Hmani et al. 2021). In another in vivo study, HME enriched in sulfated polysaccharides inhibited ROS‐induced inflammatory response in a rat experimental model (Brito et al. 2016). The antioxidant properties of HMEs have been attributed mainly to various polyphenols (e.g., epicatechin, phloretin, and tannins) (Rozo et al. 2019; Hmani et al. 2021). Moreover, compounds found by other studies in *H. musciformis* that present important bioactivities include sulfated polysaccharides (e.g., carrageenan) (das Chagas Faustino Alves et al. 2012; de Brito et al. 2013; Brito et al. 2016), ceramides (Elhady et al. 2022), tannins (Hmani et al. 2021), terpenoids (Chakraborty et al. 2016), and vitamins C and E (Balamurugan et al. 2017). Although few studies have reported the antioxidant properties of HMEs, it is crucial to comprehend the molecular mechanisms that are responsible for them, and so further research is necessary.

The use of natural antioxidants, such as compounds from seaweeds, in the form of food supplements or biofunctional foods has been suggested for the prevention and/or treatment of diseases induced by oxidative stress (Carpena et al. 2022). This preference is because seaweed compounds are natural antioxidants which are favored over synthetic ones associated with negative consequences on health for the prevention and/or treatment of oxidative stress‐induced diseases (Carpena et al. 2022). Oxidative stress, a causative factor of pathological conditions (e.g., cancer, aging, diabetes, and neurogenerative and cardiovascular diseases), is triggered by the excessive production of free radicals, mainly ROS, within cells (Curieses Andrés et al. 2023; Safaei et al. 2024; Lan et al. 2024; Rostami et al. 2023). Normal endogenous processes (e.g., oxidative energy metabolism, inflammation, and xenobiotic metabolism) in living organisms result in the production of ROS (Halliwell 2022; Amirtaheri Afshar et al. 2023; Wang, He, and Lv 2023). These ROSs play a vital role in normal cellular function, for instance, as regulatory mediators in signaling processes and defense against pathogens, when they present at appropriate low levels (Halliwell 2022). However, an excessive accumulation of ROS interacting with various biomolecules can result in oxidative stress disrupting the normal function and structure of healthy cells (Curieses Andrés et al. 2023).

For example, oxidative stress‐induced cellular damage may lead mesenchymal stem cells (MSCs) to cellular dysfunctions such as aging, dysregulating differentiation, and suppression of cell growth (Trachana et al. 2017; Huang et al. 2023). Furthermore, the utilization of MSCs has been suggested as a treatment approach for various pathologies, such as cardiovascular and neurodegenerative diseases (Goutas and Trachana 2021; Balakrishna Pillai et al. 2022; Regmi et al. 2022). Consequently, MSCs may have significant applications in the field of regenerative medicine (Goutas and Trachana 2021; Regmi et al. 2022). However, oxidative stress can cause the demise of MSCs, thereby hindering their therapeutic efficacy (Chen and Zhou 2022). Hence, it is highly significant to identify substances that can enhance MSCs' antioxidant mechanisms (Hou et al. 2022).

One of the most important antioxidant mechanisms within cells is the nuclear factor (erythroid‐derived 2)‐like 2 (NRF2) (Besednova et al. 2022; Tao et al. 2024; Zhao et al. 2023). Several seaweed compounds (e.g., sulfated polysaccharides and phlorotannins) have been shown to exert their antioxidant activity by activating NRF2 (Besednova et al. 2022). NRF2 and its endogenous inhibitor, Kelch‐like ECH‐associated protein 1 (Keap1), aim to prevent cellular homeostasis' deregulation due to oxidative stress (Bellezza et al. 2018; Miao et al. 2023). Under homeostatic conditions, as long as NRF2 is bound to Keap1, it remains inactive (Bellezza et al. 2018) and is degraded through the proteasome or the lysosome pathway via autophagy (Bellezza et al. 2018). When oxidative stress occurs, NRF2 dissociates from Keap1 and translocates into the nucleus, where it forms a transcription factor after heterodimerization with one of the Maf proteins. Once there, it acts as a transcription factor, binding to the antioxidant response element (ARE) located in the upstream promoter region of numerous antioxidant genes, thereby promoting their expression (Bellezza et al. 2018).

As mentioned above, few studies have reported that HMEs possess antioxidant properties, and consequently, HMEs have been suggested for use, for instance, as food supplements for protection against pathologies caused by oxidative stress (Rozo et al. 2019; Hmani et al. 2021). However, for this purpose, it is indispensable to elucidate the molecular mechanisms accounting for the antioxidant activity of HME's compounds. Thus, the hypothesis of the present study was that HME might exert its antioxidant activity through the induction of NRF2 pathway, one of the most important antioxidant mechanisms in the human organism. After determining HME's chemical composition and assessing its antioxidant potency in vitro, the present study investigated for the first time the involvement of the NRF2 pathway in HME's antioxidant activity in human cells.

## 2 Materials and Methods

### 2.1 *Hypnea musciformis* Collection and Extract Preparation

*H. musciformis* was collected from the Thermaikos Gulf (40°40′64.46″ N, 22°89′34.38″ E; Thessaloniki, Greece), Northern Aegean Sea, Mediterranean Sea, from June to September 2020. The identification of *H. musciformis* was made using various studies and floral catalogs (Benhissoune et al. 2003; Nauer et al. 2014; www.algaebase).

The isolation of the *H. musciformis* extract was made as described in one of our previous studies (Goutzourelas et al. 2023).

The following equation was used to assess the percentage yield of the extraction:

(1) $$\text{Extraction yield}\;\left(\%\right)=\left[\mathrm{dry}\;\text{extract}\;\left(g\right)/\mathrm{dry}\;\text{seaweed}\;\left(g\right)\right]\times 100$$

The extracts were stored at −20°C.

### 2.2 Determination of HME's Chemical Composition

#### 2.2.1 Evaluation of Total Polyphenolic Content (TPC)

The TPC value of HME was evaluated spectrophotometrically at 765 nm on a Perkin Elmer Lambda 25 UV/VIS spectrophotometer (Waltham, MA, USA) using the Folin–Ciocalteu reagent as described previously (Goutzourelas et al. 2023). Each measurement was performed in triplicate and repeated three times.

#### 2.2.2 Evaluation of Total Flavonoid Content (TFC)

The TFC of HME was measured by using the assay of AlCl_3_ according to Petrotos et al. (2021). In brief, 1.0 mL of HME (100 mg/mL in double‐distilled H_2_O_2_) was mixed in a glass tube with 3 mL of methanol, 200 μL of AlCl_3_ solution (10% w/v in water), 200 μL of potassium acetate solution (1 M), and 5.6 mL of distilled water. The mixture was incubated for 30 min at room temperature. The absorbance of each sample was assessed spectrophotometrically at 420 nm. The blank solution consisted of all the reagents, but distilled water was used instead of HME. Moreover, a negative control was used, containing all the reagents and HME, except AlCl_3_. TFC's evaluation was based on a standard curve of absorbance values correlated with standard concentrations (12.5–200 μg/mL) of quercetin. TFC was expressed as mg of quercetin equivalents (QEs) per g of dw of HME. Each measurement was performed in triplicate and on three separate occasions.

#### 2.2.3 Evaluation of Total Proteins

For evaluating HME's total proteins, the Bio‐Rad Bradford protein assay kit I (Bio‐Rad, Hercules, CA, USA) was used according to the manufacturer's instructions. Specifically, 25 μL of HME (200 mg/mL in water) was mixed with 200 μL of Bradford reagent in a 96‐well plate. The mixture was incubated at 37°C for 30 min. Then, the absorbance was assessed at 450 nm on a Perkin Elmer EnSpire Model 2300 Multilabel microplate reader (Waltham, MA, USA). A negative control consisting of the tested sample alone without Bradford reagent was used. Protein concentration was evaluated using a standard curve of absorbance values correlated with standard concentrations (125–1500 μg/mL) of bovine serum albumin (BSA). Each measurement was performed in triplicate and on three separate occasions.

#### 2.2.4 Assessment of Individual Polyphenols Using High‐Pressure Liquid Chromatography‐Diode‐Array Detection (HPLC‐DAD)

HPLC‐DAD analysis was performed to identify individual polyphenols and simple phenols in HME as described in one of our previous studies (Goutzourelas et al. 2023).

The standards included caftaric acid, caffeic acid, epigallocatechin gallate, p‐coumaric acid, chicoric acid, trans‐ferulic acid, quercetin, sinapinic acid, rutin hydrate, trans‐cinnamic acid, gallic acid, p‐hydroxyl‐benzoic acid, chlorogenic acid, vanillic acid, and myricetin (Merck, Darmstadt, Germany). The standards were dissolved in methanol and subjected to analysis at wavelengths of 280, 270, 328, and 318 nm. Each calibration curve was constructed using a mixture of standards ranging in concentration from 0.78 to 200 ppm. Phenolic content analysis of the HME was performed using a concentration of 7.0 mg/mL in methanol, and the identified compounds were compared against the standards.

#### 2.2.5 Assessment of HME's Chemical Composition Using Gas Chromatography Mass Spectrometry (GC–MS) Analysis

HME's chemical constitution was evaluated using GC–MS analysis. Specifically, 1 mg of dried extract was diluted with 200 μL methanol. For aromatic profile analysis, the sample was added to a GC–MS vial. Polar content analysis was based on a two‐step derivatization procedure performed before sample analysis. Specifically, an aliquot of 50 μL was added to a glass vial and evaporated to dryness using speedVac. Methoxymation was performed by the addition of 10 μL MeOX (methoxyamine, 40 mg/mL) at 40°C for 90 min. Then, silylation was carried out after the addition of MSTF 1% TMCS at 90°C for 30 min. Samples were left for 30 min at room temperature before analysis. Pentadecane was used as an injection standard.

Once sample was prepared, analysis was carried out on an EVOQ 456 GC‐TQ‐MS system (Bruker, Billerica, MA, USA) equipped with a CTC automatic sampler and a PTV injector, controlled by Compass Hystar software. An HP‐INNOWAX (30 m × 0.25 mm × 0.25 μm) column (Agilent Technologies, Santa Clara, CA) was used for the aromatic characterization of the sample. For derivatized samples, a 30 m HP‐5 MS UI (Agilent J&W) column (0.25 mm, ID of 0.25 μm) was used, into which 1 μL of sample was injected in a splitless mode. The carrier gas was helium (99.999%), used at a flow rate of 1.1 mL/min. For the untargeted analysis, the initial inlet temperature was 110°C for 1 min and then increased to 250°C at a rate of 250°C/min, where it was held for 12 min. The temperature then returned to the initial conditions for the remaining 24.6 min of the run. The column temperature was set to 60°C for the initial 1 min before increasing to 320°C at 10°C/min. The column then returned to the initial temperature and was held for 1 min. The total analysis time was 38 min. Electron ionization (EI) was applied, and the ion source and transfer line temperatures were set to 230°C and 250°C, respectively. Mass spectra were acquired over the range of 50–600 amu in a full scan mode, with a solvent delay of 6.8 min. Chromatographic data were processed using MSWS data processing software (Bruker Daltonics, Bremen, Germany) and the NIST17 Mass Spectral Library (mainlib library). In addition, the open‐source software pipeline MS‐DIAL (RIKEN CSRS/IMS, Tokyo, Japan) (Lai et al. 2018) was used for the identification and quantification of small molecules by mass spectral deconvolution. Chromatographic peak areas of Extracted Ion Chromatograms (EICs) together with possible identities and retention times were exported and further inspected in Microsoft Excel. The identification of most of the detected compounds was confirmed by the analysis of stable standards. For the compounds without standards, the identification probability % was calculated by the NIST17 library.

### 2.3 Free Radical Scavenging Assays

The ability of HME to scavenge free radicals in vitro was evaluated using 2,2‐diphenyl‐picrylhydrazyl radical (DPPH^·^), 2,2′‐azino‐bis(3‐ethylbenzthiazoline‐6‐sulfonic acid) radical (ABTS^·+^), hydroxyl radical (^·^OH), and superoxide anion radical (O_2_^·−^) assays. All free radical scavenging assays were performed as described in one of our previous studies (Goutzourelas et al. 2023). Ascorbic acid was used as a positive control. In all assays, the percentage of radical scavenging capacity (RSC) of the tested samples was evaluated according to the following formula:

(2) $$\mathrm{RSC}\;\left(\%\right)=\left[\left(A_{\text{control}}-A_{\text{sample}}\right)/A_{\text{control}}\right]\times 100$$

where *A*_control_ and *A*_sample_ are the absorbance values of the control and the sample, respectively. The IC_50_ value, representing the concentration at which 50% of the free radical scavenging occurred, was calculated from the graph plotted as RSC percentage against the extract concentration. In all assays, the experiments were repeated on at least three different occasions.

### 2.4 Reducing Power (RP) Assay

Reducing power was determined spectrophotometrically as described previously (Goutzourelas et al. 2023). Ascorbic acid was employed as a positive control. The RP_0.5AU_ value, indicating the extract concentration that caused an absorbance of 0.5 at 700 nm, was determined from the graph plotting absorbance against extract concentration. The experiment was repeated on at least three different occasions.

### 2.5 ROS‐Induced DNA Plasmid Strand Cleavage Assay

The ROS‐induced DNA plasmid strand cleavage assay was performed as described in one of our previous studies (Kreatsouli et al. 2019). The percentage of the inhibitory activity of HME from ROO^·^‐induced DNA strand breakage was evaluated using the following equation:

(3) $$\%\text{Inhibition}=\left[\left(S-S_{o}\right)/\left(S_{\text{control}}-S_{o}\right)\right]\times 100$$

where *S*_control_ is the percentage of supercoiled DNA in the negative control (plasmid DNA alone), *S*_o_ is the percentage of supercoiled plasmid DNA in the positive control (without the extract but in the presence of the radical initiating factor), and *S* is the percentage of supercoiled plasmid DNA in the tested extract along with the radical initiating factor. Furthermore, the IC_50_ values representing the concentration that resulted in a 50% inhibition of AAPH‐induced DNA relaxation were determined. The experiment was repeated on at least three different occasions.

### 2.6 Primary Cultures of Mesenchymal Stem Cells

Mesenchymal stem cells (WJ‐MSCs) were obtained from the Wharton Jelly of umbilical cords from term‐gestation newborns after birth, with consent obtained from the parents (three different individuals, *n* = 3) in accordance with the principles of the Declaration of Helsinki, as previously described (Tsagias et al. 2011). Isolated WJ‐MSCs were cultured as reported previously (Goutas et al. 2023). Middle passage WJ‐MSCs (15 < *p* < 40) were used for the experiments.

### 2.7 XTT Assay for Inhibition of Cell Viability

The inhibition of cell proliferation was assessed using the XTT assay kit (Roche, Germany), as described previously (Goutzourelas et al. 2023). The data were expressed as a percentage of inhibition using the following formula:
(4)

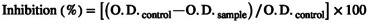

where O.D._control_ and O.D._sample_ indicate the optical densities of the negative control and the tested extract, respectively. The experiment was conducted in triplicate and repeated at least three times.

### 2.8 Cell Treatment With HME for Assessing Effects on Redox Status, DNA Damage, Gene, and Protein Expression

To evaluate HME's antioxidant activity in WJ‐MSCs', cells were seeded into 75‐cm^2^ flasks containing culture medium and incubated for 24 h at 37°C in 5% CO_2_. Different concentrations of HME dissolved in culture medium were then incubated with WJ‐MSCs for 24 h. Following this, WJ‐MSCs were collected using trypsin and used to assess lipid peroxidation, total antioxidant capacity (TAC), GSH levels, and protein oxidation. For assessing HME's ability to protect from oxidative stress, after 24‐h treatment of WJ‐MSCs with HME, the culture medium was removed and 400 μΜ of H_2_O_2_ in DMEM without FBS was added to the cell culture for 30 min.

Total ROS levels and DNA damage were also assessed in WJ‐MSCs. In these cases, although the cells were incubated with HME at 100 μg/mL and/or H_2_O_2_ as described above, they were cultured in six‐well plates (2 × 10^5^ cells per well).

The expression of antioxidant genes and proteins was also assessed in WJ‐MSCs. In these cases, the cells were treated with HME at 100 μg/mL and seeded into 25 cm^2^ flasks in the culture medium.

### 2.9 Thiobarbituric Acid Reactive Substances (TBARSs), Protein Carbonyls (CARBs), GSH, and TAC Assays

After treatment with HME and/or H_2_O_2_, the cells were detached using trypsin, and the oxidative stress markers lipid peroxidation, protein oxidation, GSH levels, and TAC were assessed using the TBARS, CARB, GSH, and TAC assays, respectively, as described previously (Kolonas et al. 2023; Kerasioti et al. 2016). Each experiment was repeated at least three times.

### 2.10 Evaluation of ROS Levels Using Immunofluorescence Microscopy

ROS levels were determined after treating WJ‐MSCs with HME at 100 μg/mL and/or H_2_O_2_ using immunofluorescence microscopy as described previously (Goutas et al. 2020). At least five randomly selected fields and a minimum of 200 cells were analyzed for each time point or condition. The means of these counts were used for the statistical analysis.

### 2.11 RNA Extraction and Quantitative Real‐Time PCR (qRT‐PCR)

qRT‐PCR analysis was performed to quantify the mRNA expression of the *NFE2L2*, *SOD1*, NAD(P)H quinone dehydrogenase 1 (*NQO1*), glutamate‐cysteine ligase catalytic subunit (*GCLC*), *GPX1*, thioredoxin (*TXN*), glutathione‐disulfide reductase (*GSR*), heme oxygenase 1 (*HMOX1*), and *CAT* genes. Initially, total cellular RNA was purified from cultured WJ‐MSCs using Trizol reagent (Invitrogen, Life Technologies, Paisley, UK) according to the manufacturer's instructions. All samples used in the study were checked for integrity and contained the 28S and 18S rRNA components. After the spectrophotometric quantification of the yield, qRT‐PCR analysis and calculations were carried out as described in our previous study (Goutas et al. 2023). Each analysis was performed in triplicates. The oligonucleotide primers are shown in Table A1 and were designed using the Primer3 input.

### 2.12 Protein Extraction and Western Blot Analysis

Protein extraction from WJ‐MSCs, Western blot analysis, and quantification of protein bands were carried out as described previously (Goutas et al. 2023). The specific primary antibodies used were against NRF2 (1:750 dilution, Cell Signaling Technology, #12721, Danvers, Massachusetts, USA), SOD1 (1:1000 dilution, Cell Signaling Technology, #4266), NQO1 (1:1000 dilution, Cell Signaling Technology, #3187), GCLC (1:1000 dilution, Cell Signaling Technology, #48005), GPX1 (1:1000 dilution, Cell Signaling Technology, #3286), TXN (1:750 dilution, Cell Signaling Technology, #2429), HMOX1 (1:1000 dilution, Cell Signaling Technology, #5853), CAT (1:1000 dilution, Cell Signaling Technology, #12980), and GSR (1:500 dilution, Santa Cruz Biotechnology, #sc‐133245). Equal protein loading was verified by reprobing each membrane with an antibody against β‐actin (1:1000 dilution, Cell Signaling Technology), which served as a loading control. Western blot analysis of subcellular fractions was performed as described previously (Goutas and Trachana 2021). Each Western blot analysis was repeated at least three times, and representative blots are shown. To calculate differences in protein expression between each time point or any other condition versus the No Treatment (NT) condition, protein expression levels at NT were arbitrary set to 1.

### 2.13 Immunofluorescence Microscopy Analysis for Assessing NRF2 Protein

Immunofluorescence microscopy analysis was used to evaluate NRF2 protein levels. The analysis was performed as described previously (Goutas and Trachana 2021). The primary antibody against NRF2 was obtained from Cell Signaling Technology (1:100, 1:750 dilution, #12721), while the appropriate fluorescent dye‐conjugated secondary antibody was obtained from Molecular Probes (1:500 dilution, Alexa Fluor 594, Molecular Probes, Eugene, OR, USA). At least five randomly selected fields and at least 200 cells were analyzed for each time point or any other condition. The means of their counts were used for the statistical analysis.

### 2.14 Subcellular Fractionation for Assessment of NRF2

NRF2 protein levels were assessed in the nuclear fraction of WJ‐MSCs to examine its activation. Thus, WJ‐MSCs at 65%–70% confluence in 25‐cm^2^ flasks were treated with HME at 100 μg/mL for 6, 12, and 24 h. Afterward, cells were collected, and pelleted cells were separated into cytoplasmic and nuclear protein fractions as described previously (Goutas and Trachana 2021). Both cytoplasmic and nuclear protein fractions were then used to assess NRF2 levels using Western blot analysis, as described above. Each experiment was performed on three separate occasions, and representative blots are shown.

### 2.15 Assessment of Protection From DNA Damage in WJ‐MSCs Using Immunofluorescence Microscopy

γ‐H2AX and 53BP1 protein levels were evaluated in WJ‐MSCs to determine DNA double‐strand breaks (DSB). Thus, after treatment of WJ‐MSCs with HME at 100 μg/mL and/or H_2_O_2_, γ‐H2AX and 53BP1 proteins were assessed using immunofluorescence microscopy, as described previously (Goutas and Trachana 2021).

### 2.16 Statistical Analysis

All results were expressed as mean ± standard error (mean ± SE). The statistical analysis was based on one‐way ANOVA followed by Dunnett's test for multiple pairwise comparisons. Differences were considered significant at *p* < 0.05. SPSS software (version 14.0; SPSS) was used for all statistical analyses.

## 3 Results

### 3.1 Determination of HME's Extraction Yield and Chemical Composition

The extraction yield for HME was 23.6% (Table 1). The HME's TPC value, as evaluated by the Folin–Ciocalteu assay, was 1.79 mg GAE/g dw extract, while TFC was 1.45 mg QE/g dwe extract (Table 1). In addition, HME contained 5.3 mg/g dw extract of total proteins (Table 1).

**TABLE 1:** Total polyphenols (TPC), total flavonoids (TFC), total proteins, and extraction yield of HME.

	TPC^a^ mg GAE/g dw Extract	TFC^a^ mg QE/g dw Extract	Total proteins^a^ mg/g dw Extract	Extraction yield^a^ (%)
*H. musciformis* extract	1.79 ± 0.02	1.45 ± 0.03	5.3 ± 0.01	23.6 ± 1.2

^a^ Values are the mean ± SEM of at least three separate measurements.

Moreover, the identification of 15 individual polyphenols was performed using HPLC‐DAD analysis. The results exhibited that none of the examined polyphenols were present in HME (Figure A1).

**FIGURE 1:**
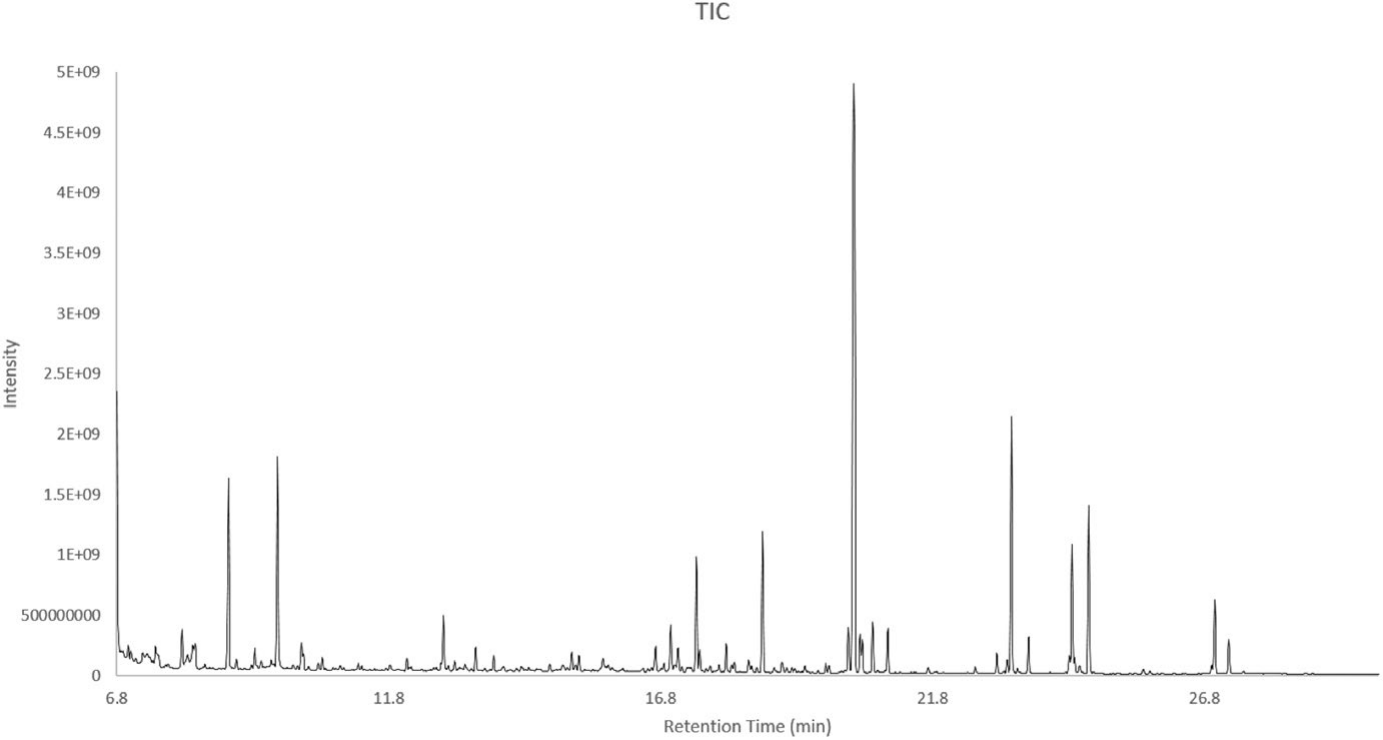
GC–MS total ion chromatogram (TIC) of the derivatized sample. For polar content analysis, a two‐step derivatization procedure was performed before sample analysis as described in Section 2.

Also, GC–MS analysis showed a poor profile of aromatic volatile components compared to the analysis of the derivatized sample, which provided a rich profile of polar components as well as more information about the constituents (Table 2; Figure 1). Thus, a total of 26 compounds from the identified peaks were of interest for this particular study and are presented in Table 2. Most of the detected compounds had an MS 1 confidence level of identification (Table 2). For the compounds that did not have standards for their identification, the identification probability percentage was calculated using the NIST17 library (Table 2). Specifically, the identification of the derivatized samples revealed that HME contained amino acids (i.e., L‐alanine, glycine, L‐valine, L‐isoleucine, serine, *β*‐alanine), organic acids (i.e., lactic acid, 3‐hydroxybutyric acid, 4‐Hydroxybutanoic acid, butanedioic acid, D‐gluconic acid), organic amides (i.e., urea), sugar alcohols [i.e., L‐(−)‐arabitol, D‐mannitol, myo‐inositol], amines (i.e., cadaverine, tyramine, ethanolamine), saturated fatty acids (i.e., myristic acid, stearic acid, hexadecanoic acid), fatty acid esters [i.e., hexadecanoic acid, methyl ester, octadecenoic acid (Z)‐, methyl ester, 2‐palmitoylglycerol], hydrogenated diterpene alcohols (i.e., phytol), and other organic compounds (i.e., glycerol‐3‐galactoside, 25‐OH cholesterol) (Table 2).

**TABLE 2:** Compounds identified in HME from the GC analysis of derivatized samples.

Compounds	Rt (min)	MF	MW	Identification	
				Confidence level	Probability (%)
Lactic acid	6.91	C_3_H_6_O_3_	90.08	1	
L‐alanine	7.28	C_13_H_18_C_l2_N_2_O_2_	305.2	1	
3‐Hydroxybutyric acid	8.24	C_4_H_8_O_3_	104.1	1	
L‐valine	9.00	C_5_H_11_NO_2_	117.15	1	
4‐Hydroxybutanoic acid	9.28	C_4_H_8_O_3_	104.1		83.0
Urea	9.34	CH_4_N_2_O	60.056		95.8
Ethanolamine	9.64	C_2_H_7_NO	61.08	1	
L‐isoleucine	10.04	C_6_H_13_NO_2_	131.17	1	
Glycine	10.23	C_2_H_5_NO_2_	75.07	1	
Butanedioic acid	10.33	C_4_H_6_O_4_	118.09		45.3
Serine	10.91	C_3_H_7_NO_3_	105.09	1	
*β*‐alanine	11.82	C_3_H_7_NO_2_	89.093		80.9
L‐(−)‐Arabitol	15.24	C_5_H_12_O_5_	152.15		40.6
Cadaverine	16.57	C_5_H_14_N_2_	102.18	1	
Myristic acid	16.71	C_14_H_28_O_2_	228.37	1	
Tyramine	17.36	C_8_H_11_NO	137.18	3	
D‐mannitol	17.46	C_6_H_14_O_6_	182.17	1	
Hexadecanoic acid, methyl ester	17.52	C_17_H_34_O_2_	270.5	1	
D‐gluconic acid	18.15	C_6_H_12_O_7_	196.16	1	
Myo‐inositol	19.04	C_6_H_12_O_6_	180.16	1	
Octadecenoic acid (Z)‐, methyl ester	19.22	C_19_H_36_O_2_	296.5	1	
Phytol	19.84	C_20_H_40_O	296.5		93.6
Stearic acid	20.47	C_18_H_36_O_2_	284.5	1	
Glycerol‐3‐galactoside 2	20.98				10.5
2‐Palmitoylglycerol	22.99	C_19_H_38_O_4_	330.5		60.7
25‐OH cholesterol	26.99	C_27_H_46_O_2_	402.7		48.5

Abbreviations: MF, molecular formula; MW, molecular weight; Rt, retention time.

### 3.2 Assessment In Vitro of Free Radical Scavenging, Reducing Power and Protection From ROS‐Induced DNA Damage

**FIGURE 2:**
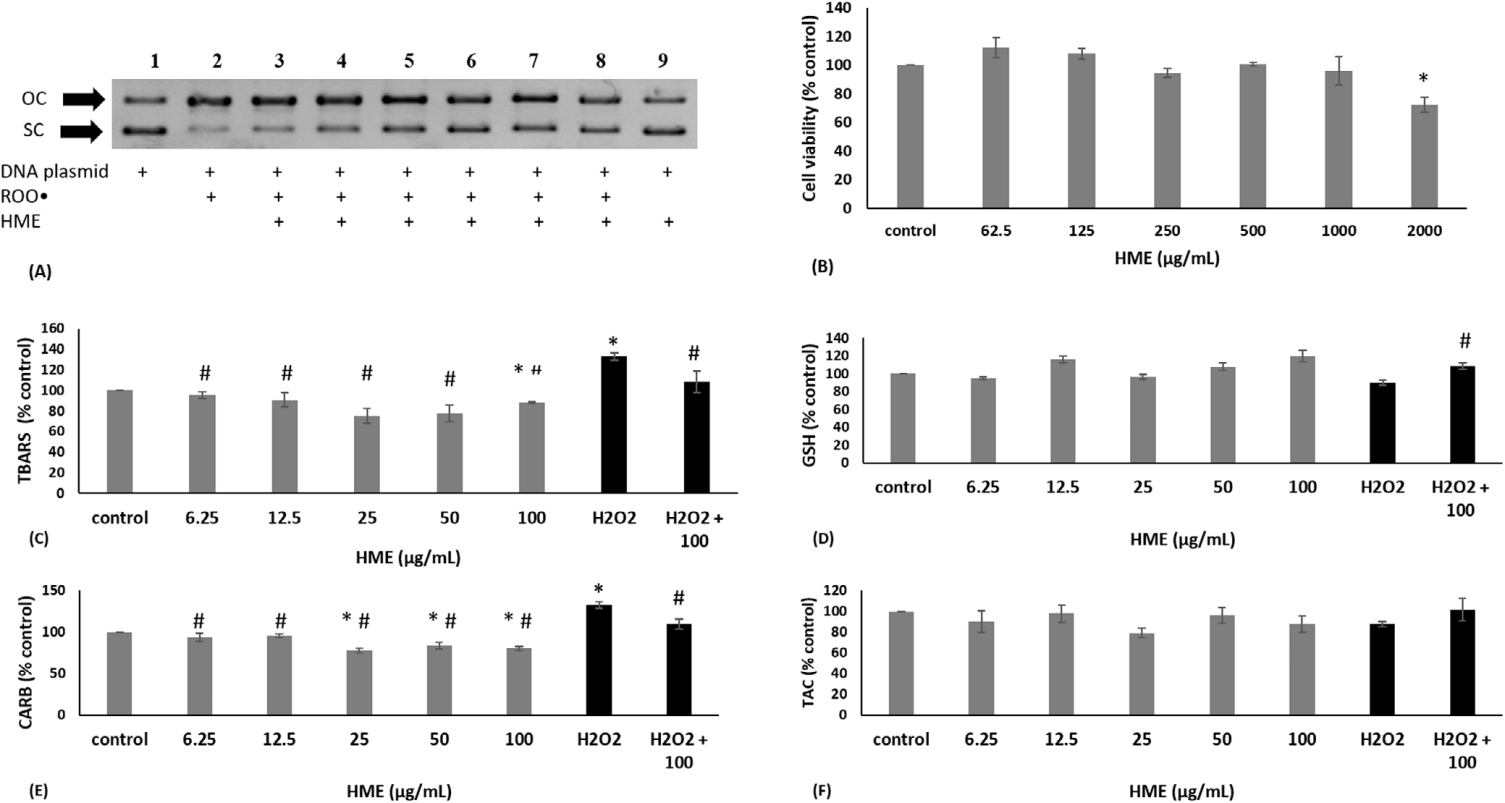
(A) Representative DNA plasmid gel electrophoresis image showing in vitro the protective activity of ΗΜΕ against ROO^·^‐induced DNA damage. OC: open circular; SC: supercoiled. (B) Cell viability following the treatment of WJ‐MSCs with HME. (C–F) Effects of HME on TBARS, CARB, GSH, and TAC levels in WJ‐MSCs in the absence and/or presence of H_2_O_2_. All values are presented as the mean ± SEM of three independent experiments. *Statistically significant compared to control (*p* < 0.05). ^#^Statistically significant compared to H_2_O_2_‐alone treatment (*p* < 0.05).

The HME demonstrated in vitro scavenging activity against the examined free radicals, DPPH^*·*^, ABTS^*·*+^, ^*·*^OH, and O_2_^*·*−^, with IC_50_ values of 12.20, 1.17, 0.74, and 0.4 mg/mL, respectively (Table 3). Moreover, HME exhibited reducing activity, with an RP_0.5AU_ value of 3.03 mg/mL (Table 3). Finally, HME protected in vitro from ROO^*·*^‐induced DNA damage with an IC_50_ value of 1.08 mg/mL (Table 3; Figure 2A).

**TABLE 3:** Free scavenging against DPPH^·^, ABTS^·+^, OH^·^, and O_2_^·*−*^ radicals, reducing power (RP) activity and protective activity against peroxyl radical (ROO^·^)‐induced DNA damage of HME.

	IC_50_ (mg/mL)					RP_0.5AU_ (mg/mL)
	DPPH^·a^	ABTS^·+^ ^a^	^·^OH^a^	O_2_^·−^ ^a^	ROO^·^ ^a^	RP^a^
*H. musciformis* extract	12.20 ± 0.50*	1.17 ± 0.07*	0.74 ± 0.01*	0.4 ± 0.01*	1.08 ± 0.02*	3.03 ± 0.26
Positive control						
Ascorbic acid	0.004 ± 0.0001*	0.003 ± 0.0001*	0.230 ± 0.015*	ND	NT	0.004 ± 0.017*

Abbreviations: ND, not determined IC_50_ values (i.e., ascorbic acid could not achieve 50% inhibition at the tested concentrations); NT, not tested.

^a^ Values are the mean ± SD of at least three separate experiments.

* *p* < 0.05 indicates significant difference from the control.

### 3.3 Cell Viability Assay

For examining HME's antioxidant activity in cells, noncytotoxic concentrations were selected by assessing its effects on cell viability using the XTT assay. The results showed that HME had significant cytotoxicity in WJ‐MSCs at concentrations higher than 2 mg/mL (Figure 2B). Thus, noncytotoxic concentrations, ranging from 6.25 to 100 μg/mL, were used for all the following assays.

### 3.4 Effects of HME on Oxidative Stress Markers in WJ‐MSCs

**FIGURE 3:**
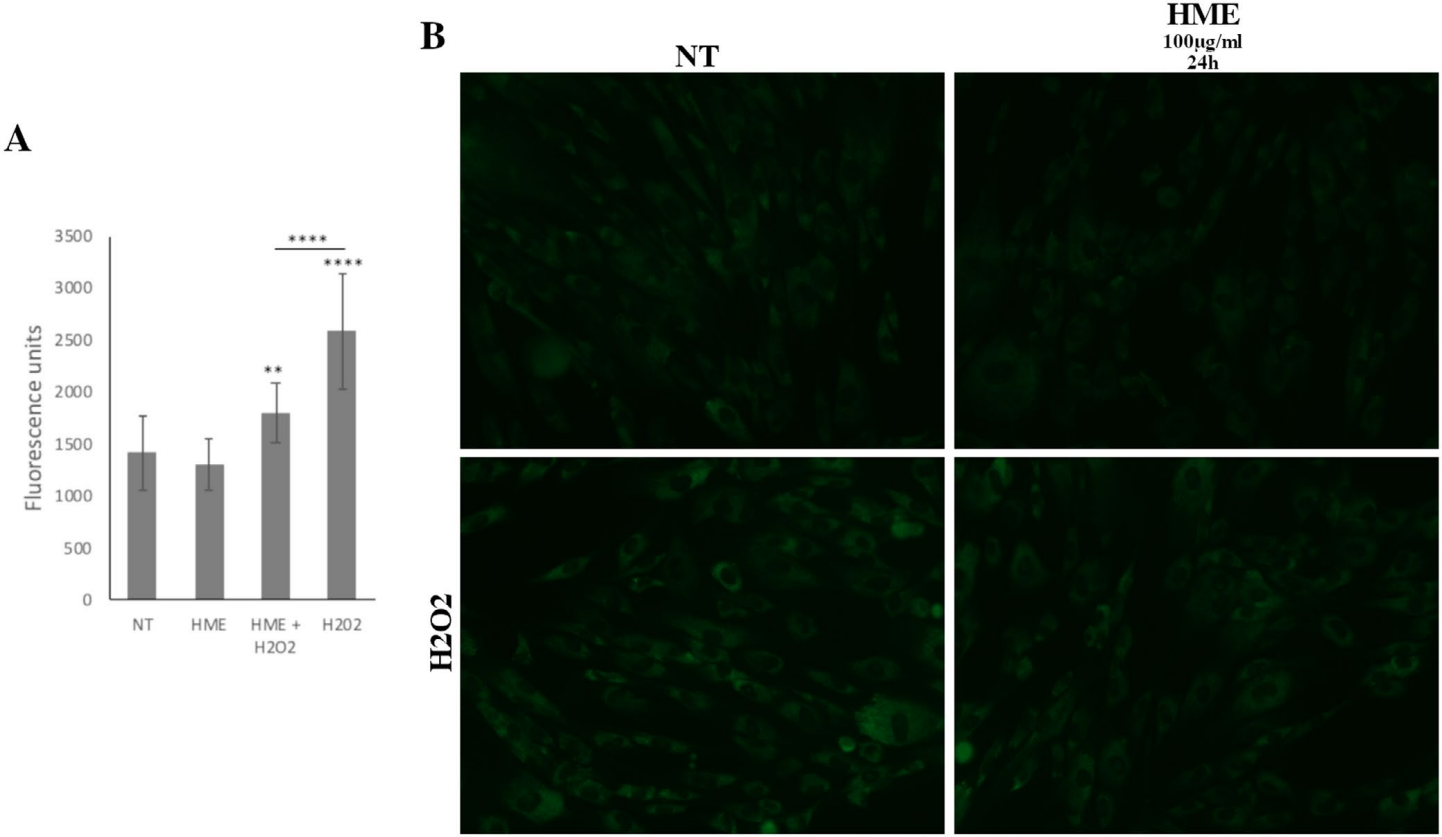
The protection of HME at 100 μg/mL against induction of ROS levels by acute oxidative stress (i.e., H_2_O_2_ treatment) in WJ‐MSCs. (A) Graph showing the fluorescence units indicating total ROS levels of each condition. ROS levels were analyzed in four conditions: (i) not treated cells (NT), (ii) cells treated with HME alone for 24 h, (iii) cells treated with HME for 24 h followed by H_2_O_2_ treatment for 30 min, and (iv) cells treated with H_2_O_2_ for 30 min. Values shown are the means ± SEM. **p < 0.01, ****p < 0.0001 versus the NT condition or otherwise indicated. (B) Representative images of WJ‐MSCs of each condition where total ROS levels were analyzed. Images were captured with the 40× objective lens of the fluorescent microscope used.

The results demonstrated that treatment with HME alone decreased significantly TBARS by 11.75% of WJ‐MSCs at 100 μg/mL, compared to control (Figure 2C). Moreover, in WJ‐MSCs treated with HME, CARB levels were significantly reduced by 22.30%, 16.22%, and 19.54% at 25, 50, and 100 μg/mL, respectively, compared to control (Figure 2E). GSH, TAC, and ROS levels were not significantly changed by treatment with HME alone in WJ‐MSCs, compared to control (Figures 2D,F and 3A,B).

Furthermore, when WJ‐MSCs were initially treated with HME at 100 μg/mL for 24 h and then subjected to oxidative stress, there was a decrease in TBARS by 24.62%, CARB by 23.08%, and ROS by 31.70%, while GSH levels were increased by 19.32%, compared to treatment with H_2_O_2_ alone (Figures 2C–E and 3A,B). The HME treatment did not significantly affect TAC in WJ‐MSCs in the presence of H_2_O_2_ (Figure 2F).

### 3.5 Effects of HME on NRF2 Activation in WJ‐MSCs

**FIGURE 4:**
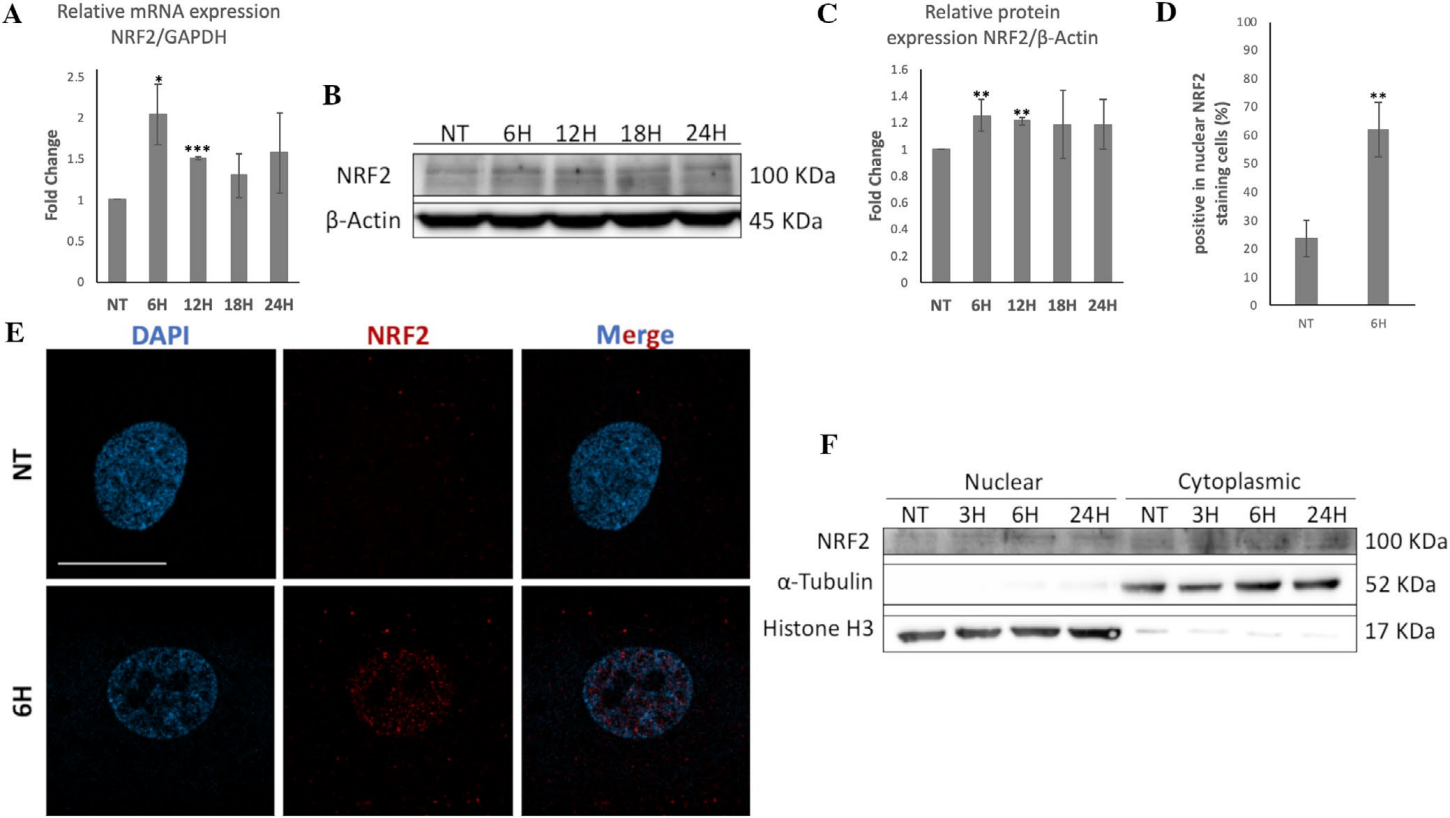
The activation of NRF2 in WJ‐MSCs treated with HME at 100 μg/mL. (A) Graph showing the alterations in NFE2L2's mRNA levels in WJ‐MSCs after HME treatment at four different time points. qRT‐PCR was used to evaluate mRNA levels, and relative levels were expressed as fold of not treated cells (NT) after normalization to GAPDH. (B) Representative immunoblot of cell lysates of WJ‐MSCs treated with HME for four different time points using antibody against NRF2. β‐Actin was used as loading control. (C) Graph showing the protein levels of NRF2 in WJ‐MSCs treated with HME for four different time points. Value of NT was arbitrary set to 1. (D) Graph showing the percentage of cells positive in nuclear NRF2 staining in NT and cells treated with HME for 6 h. (E) Representative images of WJ‐MSCs of NRF2 staining in NT and cells treated with HME for 6 h. Nuclei were stained with DAPI. Images were captured with the 66× objective lens of the confocal fluorescent microscope. (F) Representative immunoblot of cell lysates of WJ‐MSCs treated with HME for three different time points after subcellular fractionation to cytoplasmic and nuclear fractions using the anti‐NRF2 antibody. α‐Tubulin was used as loading control for cytoplasmic fractions, and Histone H3 was used as loading control for nuclear fractions. Values shown are the means ± SEM. *p < 0.05, **p < 0.01, ***p < 0.001 versus the NT condition.

HME treatment significantly upregulated the mRNA levels of the *NFE2L2* gene encoding for NRF2, by 2‐fold and 1.5‐fold at 6 and 12 h, respectively, compared to control (Figure 4A). Moreover, NRF2 protein levels were also significantly increased in WJ‐MSCs, after HME treatment for 6 and 12 h, by 25% and 21%, respectively, compared to control (Figure 4B,C). Furthermore, the immunofluorescence analysis revealed that HME treatment for 6 h promoted significantly greater NRF2 translocation to the nucleus of WJ‐MSCs compared to control. Specifically, 62% of HME‐treated cells showed NRF2 translocation to the nucleus versus 23.6% of untreated cells (Figure 4D,E). The HME‐induced NRF2's nuclear translocation was further supported by an increase in NRF2 levels in the nuclear fraction of WJ‐MSCs after HME treatment for 6 h compared to control (Figure 4F).

### 3.6 Effects of HME on the Expression of Antioxidant Genes in WJ‐MSCs

**FIGURE 5:**
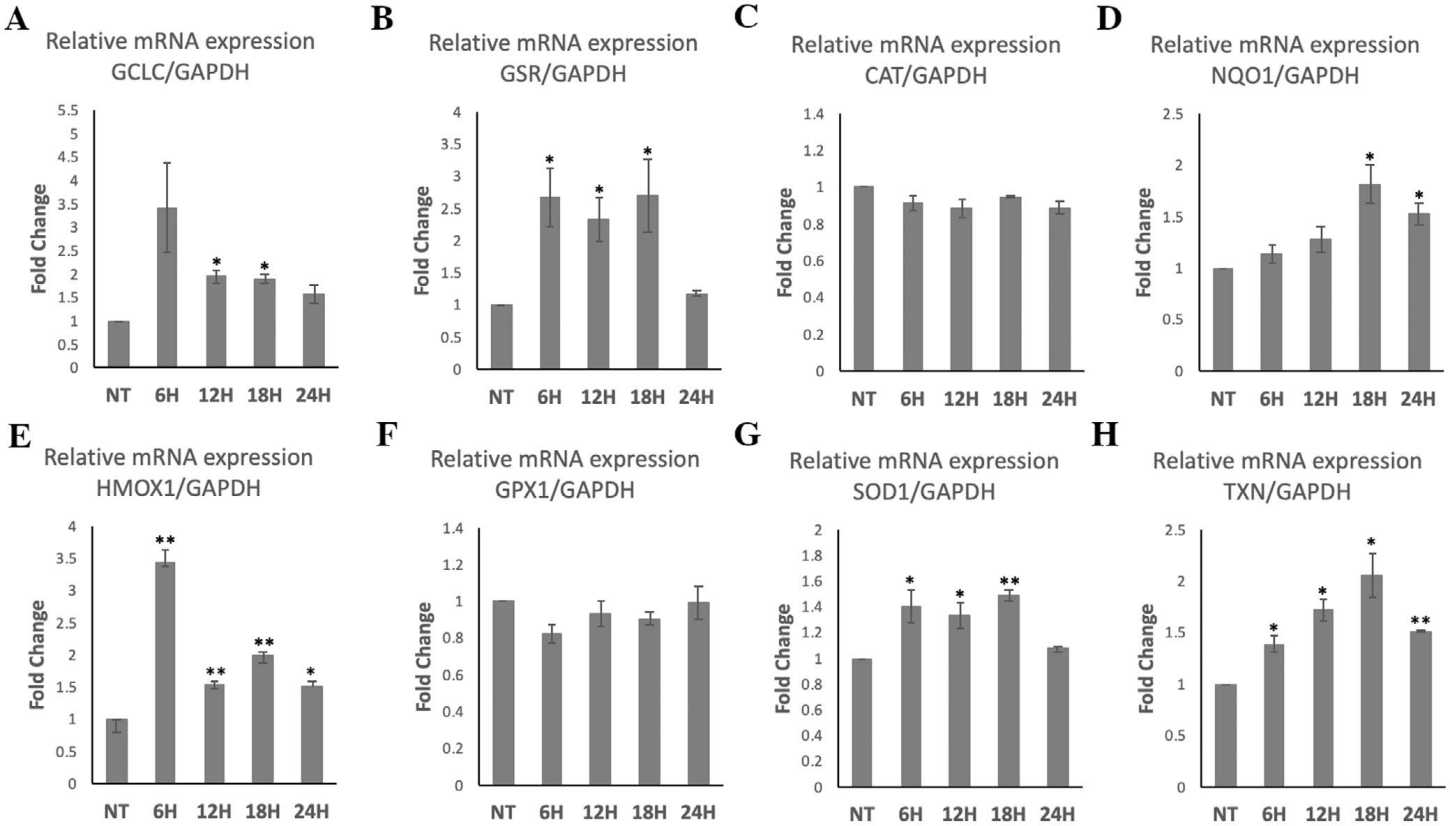
Gene expression profiles of GCLC, GSR, CAT, NQO1, HMOX1, GPX1, SOD1, and TXN genes in WJ‐MSCs after HME treatment at 100 μg/mL for four different time points. mRNA levels were determined by qRT‐PCR, and relative levels were expressed as fold of NT (not treated cells) after normalization to GAPDH. Value of NT was arbitrary set to 1. The results are expressed as means ± SEM. *p < 0.05, **p < 0.01 versus the NT condition.

HME treatment significantly increased the mRNA levels of *GCLC* by 1.95‐fold and 1.9‐fold at 12 and 18 h, respectively; *GSR* by 2.66‐fold, 2.33‐fold, and 2.69‐fold at 6, 12, and 18 h, respectively; *NQO1* by 1.81‐fold and 1.53‐fold at 18 and 24 h, respectively; *HMOX1* by 3.42‐fold, 1.54‐fold, 1.97‐fold, and 1.49‐fold at 6, 12, 18, 24 h, respectively; *SOD1* by 1.40‐fold, 1.33‐fold, and 1.49‐fold at 6, 12, and 18 h, respectively; and *TXN* by 1.38‐fold, 1.71‐fold, 2.05‐fold, and 1.51‐fold at 6, 12, 18, and 24 h, respectively, compared to control (Figure 5A,B,D,E,G,H). HME treatment did not significantly change the mRNA levels of *CAT* and *GPX1* compared to control (Figure 5C,F).

**FIGURE 6:**
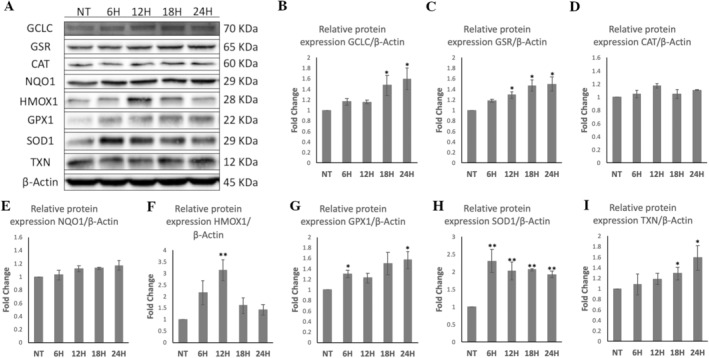
Protein expression profiles of GCLC, GSR, CAT, NQO1, HMOX1, GPX1, SOD1, and TXN in WJ‐MSCs after HME treatment at 100 μg/mL. (A) Representative immunoblot of cell lysates of WJ‐MSCS treated with HME for four different time points using antibodies against the tested proteins. Protein levels were determined by Western blot, and relative levels were expressed as fold of NT (not treated cells) after normalization to β‐actin. Graphs showing the (B) GCLC, (C) GSR, (D) CAT, (E) NQO1, (F) HMOX1, (G) GPX1, (H) SOD1, and (I) TXN protein levels. Value of NT was arbitrary set to 1. The results are expressed as means ± SEM. *p < 0.05, **p < 0.01 versus the NT condition.

Furthermore, HME treatment significantly increased the protein levels of GCLC by 1.47‐fold and 1.59‐fold at 18 and 24 h, respectively; GSR by 1.29‐fold, 1.46‐fold, and 1.49‐fold at 12, 18, and 24 h, respectively; HMOX1 by 3.12‐fold at 12 h; GPX1 by 1.3‐fold and 1.56‐fold at 6 and 24 h, respectively; SOD1 by 2.3‐fold, 2.01‐fold, 2.06‐fold, and 1.92‐fold at 6, 12, 18, and 24 h, respectively; and TXN by 1.28‐fold and 1.58‐fold at 18 and 24 h, respectively (Figure 6A–C,F–I). HME treatment did not significantly change CAT and NQO1 levels compared to control (Figure 6A,D,E).

### 3.7 Effects of HME on the Protection of ROS‐Induced DSBs in WJ‐MSCs

**FIGURE 7:**
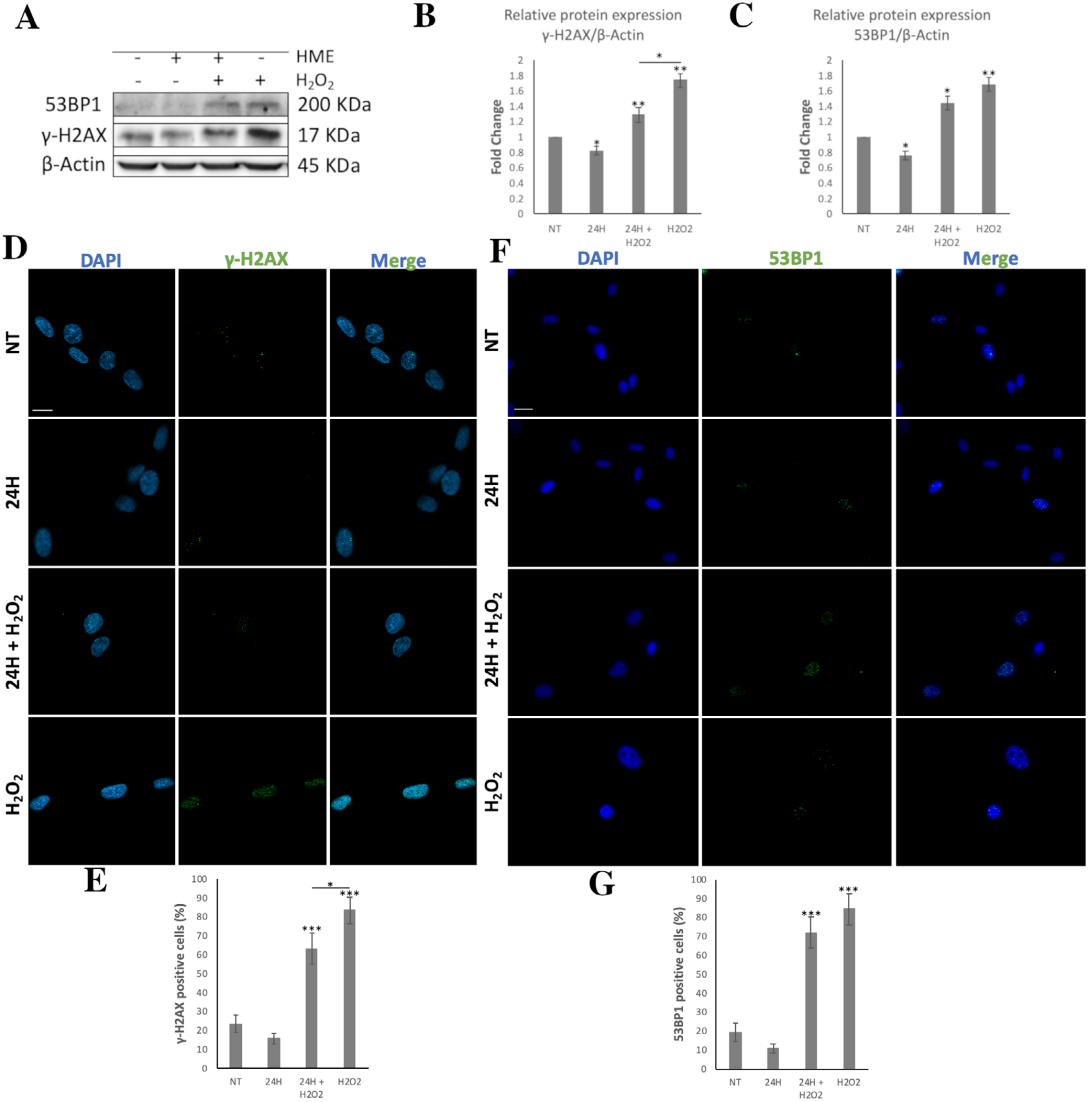
The protection of HME at 100 μg/mL from DSBs induced by acute oxidative stress (i.e., H_2_O_2_ treatment) in WJ‐MSCs. Cells were subjected to four conditions: (i) not treated cells (NT), (ii) cells treated with HME alone for 24 h, (iii) cells treated with HME for 24 h followed by H_2_O_2_ treatment for 30 min, and (iv) cells treated with H_2_O_2_ for 30 min. (A) Representative immunoblot of cell lysates of WJ‐MSCs using antibodies against γ‐Η2ΑΧ and 53BP1 proteins. β‐Actin was used as loading control. Graphs showing the (B) γ‐Η2ΑΧ and (C) 53BP1 protein levels. Value of NT was arbitrary set to 1. Representative images of WJ‐MSCs of (D) γ‐Η2ΑΧ and (F) 53BP1 staining. Nuclei were stained with DAPI. Images were captured with the 40× objective lens of the confocal fluorescent microscope and fluorescent microscope used for γ‐Η2ΑΧ and 53BP1 assessment, respectively. Graphs showing the percentage of cells positive in (E) γ‐Η2ΑΧ and (G) 53BP1 staining. The results are expressed as means ± SEM. *p < 0.05, **p < 0.01, ***p < 0.001 versus the NT condition or otherwise indicated.

The results of the immunofluorescence analysis showed that when WJ‐MSCs were treated with HME for 24 h before H_2_O_2_ treatment, the percentage of γ‐H2AX positive cells was significantly decreased by 20.3% compared to cells treated only with H_2_O_2_ (Figure 7D,E). There was also a decrease, although not statistically significant, in the percentage of 53BP1 positive cells in WJ‐MSCs treated with HME for 24 h before H_2_O_2_ treatment compared to cells treated only with H_2_O_2_ (Figure 7F,G). Likewise, the reduction in 53BP1 and γ‐H2AX‐positive cells was not significant between HME alone‐treated WJ‐MSCs and untreated cells (Figure 7D–G).

Furthermore, the protein levels of γ‐H2AX were significantly reduced by 25.86% in cells that were treated with HME before H_2_O_2_ treatment compared to cells treated only with H_2_O_2_ (Figure 7A,B). Similar to the immunofluorescence analysis, there was a nonsignificant decrease in 53BP1 in WJ‐MSCs treated with HME for 24 h before H_2_O_2_ treatment compared to cells treated only with H_2_O_2_ (Figure 7A,C). However, interestingly, the protein levels of γ‐H2AX and 53BP1 were decreased significantly by 18.00% and 24.00%, respectively, in HME‐treated cells compared to control (i.e., untreated cells) (Figure 7A–C).

## 4 Discussion

The extraction yield of HME was 23.6%, which was similar to that of other seaweed species extracts isolated with the same method in one of our previous studies (Goutzourelas et al. 2023). Chakraborty, Joseph, and Praveen (2015) reported a yield of 4.83% of methanolic extract from *H. musciformis* , but the isolation process was different from our method.

TPC was low, but it was close to what found in other studies (Vatan, Celikler, and Yildiz 2011; Rozo et al. 2019; Pangestuti et al. 2019). However, other studies showed higher TPC values of *H. musciformis* extracts than our value (Hmani et al. 2021; Chakraborty, Joseph, and Praveen 2015; Pangestuti et al. 2019; Arulkumar et al. 2020). The difference between other studies' TPC values and our value may be due to differences in seaweeds' geographical location (Chakraborty, Joseph, and Praveen 2015; Arulkumar et al. 2020; Hmani et al. 2021) and/or extraction method (Chakraborty, Joseph, and Praveen 2015; Pangestuti et al. 2019; Arulkumar et al. 2020). Moreover, the results showed that a large part (i.e., the 80%) of HME's polyphenols were flavonoids. Hmani et al. (2021) found a TFC value in HME that was similar to our value. While Arulkumar et al. (2020) reported higher TFC compared to our value, but again flavonoids constituted the majority (about 95%, of polyphenols). We also tried to identify 15 individual polyphenols in HME using HPLC‐DAD analysis, but none of them were detected. Apart from gallic acid and chlorogenic acid (Pangestuti et al. 2019), there has not been any study identifying these polyphenols in *H. musciformis*, although they were found in other seaweed species (Santos et al. 2019; Yuan et al. 2019; Aminina et al. 2020). Moreover, other polyphenols such as protocatechuic acid (Pangestuti et al. 2019), (−)‐epicatechin, and phloretin (Rozo et al. 2019) have been found in HMEs.

Proteins and peptides from seaweeds including *H. musciformis* exhibited antioxidant properties (Pangestuti et al. 2019; Varona et al. 2023), and so the total proteins of HME were evaluated. The total protein amount was 5.3 mg/g dw of extract. In other studies, the total protein content of HMEs varied from 10 to 400 mg/g dw (Vatan, Celikler, and Yildiz 2011; das Chagas Faustino Alves et al. 2012; Pangestuti et al. 2019). The great variation in protein content of seaweed extracts between different studies has been attributed to various experimental (e.g., extraction and purification procedures), environmental, seasonal, and geographical factors (Carpena et al. 2022).

Moreover, the GC–MS analysis identified various compounds in HME, such as the two essential amino acids L‐valine and L‐isoleucine and the four nonessential amino acids L‐alanine, glycine, serine, and *β*‐alanine. Dried sample of *H. musciformis* from the Indian coast has been reported to contain all of these amino acids along with others (Shareef Khan, Sridharan, and Abdul Nazar 2012). In addition, HME contained the saturated fatty acids, myristic acid, stearic acid, and hexadecanoic acid. All of these have been found previously in *H. musciformis* (Shareef Khan, Sridharan, and Abdul Nazar 2012; Rafiquzzaman et al. 2015). Moreover, esters of fatty acids were detected in HME for the first time, specifically hexadecanoic acid, methyl ester, octadecenoic acid (Z)‐, methyl ester, and 2‐palmitoylglycerol. Our chemical analysis also identified for the first time in HME the organic acids lactic acid, 3‐hydroxybutyric acid, 4‐hydroxybutanoic acid, butanedioic acid, and D‐gluconic acid, the organic amide urea, the sugar alcohols L‐(−)‐arabitol, D‐mannitol and myo‐inositol, the amines cadaverine, tyramine and ethanolamine, the hydrogenated diterpene alcohol phytol, and the organic compounds glycerol‐3‐galactoside and 25‐OH cholesterol. Several of the compounds identified in HME have been reported to possess antioxidant activity, as summarized in Table 4.

**TABLE 4:** Compounds identified in HME from the chemical analysis of the present study have been demonstrated to possess antioxidant activity.

Compounds identified in HME	Antioxidant activity	References
Polyphenols (e.g., phloretins, epicatechin)	Induced NRF2; exhibited antioxidant capacity	Ruan et al. (2023), Pangestuti et al. (2019), Rozo et al. (2019)
Serine	Induced NRF2	Maralani, Movahedian, and Javanmard (2012)
Lactic acid	Inhibited ROS in neural cells	Lampe et al. (2009)
β‐alanine	Increased TAC and GSH and decreased ROS in human plasma	De França et al. (2019)
Glycine	Precursor molecule for GSH synthesis, decreased lipid peroxidation, and enhanced GSH synthesis	Razak et al. (2017), Senthilkumar, Sengottuvelan, and Nalini (2004)
Valine	Alleviated oxidative stress in rats	Cojocaru et al. (2014)
Isoleucine	Decreased H_2_O_2_‐induced ROS increase by increasing TAC and reducing oxidation of lipids in bovine mammary epithelial cell lines	Wu et al. (2022)
3‐Hydroxybutyric acid	Inhibited H_2_O_2_‐induced oxidative stress through augmentation of TXN in rat cardiomyocytes	Wang et al. (1999)
Tyramine	Inhibited in vitro lipid peroxidation, scavenged ^·^OH and O_2_^·−^ radicals, and exhibited reducing activity	Yen and Hsieh (1997)
Mannitol	Protected different tissues from oxidative stress induced by various factors (e.g., UV, H_2_O_2_) in in vivo and cell culture experiments	André and Villain (2017)
Myo‐inositol	Increased CAT, GPX, GR, GSH, and SOD and decreased TBARS and CARB in different Jian carp tissues	Jiang et al. (2009), López‐Gambero et al. (2020)
Octadecenoic acid (Z)‐, methyl ester	Exhibited antioxidant capacity	Elwekeel et al. (2023)
Phytol	Inhibited TBARS formation and scavenge ^·^OH and NO	Santos et al. (2013)
Stearic acid	Protected rat cortical neurons from H_2_O_2_‐induced oxidative damage and mitigated oxidation of lipids through enhancement of CAT and SOD	Wang et al. (2007)

Furthermore, the results showed that HME could scavenge in vitro DPPH^**·**^, ABTS^**·**+^, OH^**·**^, and O_2_^**·** *−*^ radicals. It was worth mentioning that HME's antioxidant compounds scavenged less effectively DPPH^**·**^ than the other radicals. Given that methanol and water are the solvents for DPPH^**·**^ and all other assays, respectively, HME's hydrophilic compounds may mainly account for its free radical scavenging activity. Both O_2_^**·** *−*^ and ^**·**^OH can cause oxidative damage to biomolecules within cells, making their scavenging an important antioxidant defense mechanism (Zeng et al. 2023). Compared to other studies, Hmani et al. (2021) and Chakraborty, Joseph, and Praveen (2015) found similar IC_50_ values of HME in free radical scavenging assays to ours (i.e., 13.9 mg/mL in DPPH^**·**^; 1.5 mg/mL in both ABTS^**·**+^ and ^**·**^OH). However, two studies reported lower IC_50_ values (about 1.0 and 3.0 mg/mL) of HME in the DPPH^**·**^ assay than our value (Chakraborty, Joseph, and Praveen 2015; Rafiquzzaman et al. 2015). Since they used a similar extraction method to ours, this difference may be attributed to variations in the extracts' chemical composition due to environmental and geographical factors (Carpena et al. 2022). Interestingly, a sulfated polysaccharide extract from *H. musciformis* exhibited much higher IC_50_ values than ours in the DPPH^**·**^, OH^**·**^, and O_2_^**·** *−*^ assays (das Chagas Faustino Alves et al. 2012), indicating that sulfated polysaccharides are not the major antioxidant compounds of this seaweed. On the other hand, terpenoids isolated from ethyl acetate extract of *H. musciformis* showed strong activity in the DPPH^**·**^ assay (Chakraborty et al. 2016). The HME's scavenging activity of free radicals was also supported by its reducing properties shown in the RP assay. As is known, free radicals are neutralized when they receive electrons (Halliwell 2022).

In addition, HME treatment of even naïve WJ‐MSCs decreased significantly lipid peroxidation and protein oxidation compared to controls. Moreover, treatment of WJ‐MSCs with HME before their exposure to H_2_O_2_ decreased ROS and consequently TBARS and CARB, compared to cells treated with H_2_O_2_ alone. These findings indicated that HME treatment protected WJ‐MSCs from oxidative stress damage. The protection of WJ‐MSCs from lipid peroxidation is important, since the latter can result in harmful effects on various cellular components, particularly cellular membranes, ultimately leading to cellular death (Gaschler and Stockwell 2017). There is also evidence that lipid peroxidation could lead to stem cell senescence (Papsdorf and Brunet 2019). Likewise, protection by HME from protein oxidation in cells is significant since protein modifications by ROS may alter their roles as enzymes, receptors, and structural components (Kehm et al. 2021). Moreover, oxidative proteins were shown to accumulate in aged human MSCs (Cheng et al. 2023). In addition, treatment of WJ‐MSCs with HME before their exposure to oxidative stress increased GSH, compared to cells exposed only to H_2_O_2_. This result suggested that HME's protection of WJ‐MSCs against oxidative stress was due, at least in part, to the enhancement of the GSH system, one of most important antioxidant mechanisms (Haddad et al. 2021). Intriguingly, HME treatment did not affect TAC, a marker of the total nonenzymatic antioxidant molecules in cells. This may be due to HME‐induced increase of some antioxidant molecules (e.g., GSH) in WJ‐MSCs, while at the same time, as a compensation effect, some other antioxidants were decreased (Alvarez and Boveris 2000), and so TAC was not changed. The present results demonstrated for the first time HME to inhibit protein oxidation. However, our results are in accordance with previous studies exhibiting that HMEs inhibited lipid peroxidation in vitro (das Chagas Faustino Alves et al. 2012; Chakraborty et al. 2016; Rozo et al. 2019) as well as in vivo in plasma, erythrocytes, and intestinal tissue of rats (Brito et al. 2016; Balamurugan et al. 2017). Furthermore, HME administration to rats has been reported to increase GSH in erythrocytes and mammary and intestinal tissues (Brito et al. 2016; Balamurugan et al. 2017).

HME was also shown to inhibit in vitro ROO^**·**^‐induced DNA plasmid strand breakage. Similar to our result, HME has previously been shown to protect in vitro from OH^**·**^‐induced DNA breakage (Rafiquzzaman et al. 2015). Our finding was further confirmed at the cellular level, when HME treatment of WJ‐MSCs before their exposure to H_2_O_2_ reduced both γ‐H2AX positive cells and γ‐H2AX protein levels compared to cells exposed only to H_2_O_2_. γ‐H2AX formation is considered an exceptionally specific and sensitive molecular marker for monitoring the initiation and resolution of DNA's DSBs (Mah, El‐Osta, and Karagiannis 2010). Nevertheless, HME treatment of WJ‐MSCs before their exposure to H_2_O_2_ did not significantly decrease 53BP1 protein compared to cells treated only with H_2_O_2_. In mammalian cells, 53BP1 is also considered a marker of DNA damage since it is a crucial molecule in the signaling and repair processes of DNA's DSBs (Panier and Boulton 2014). However, γ‐H2AX is more significant than 53BP1 for detecting DNA's DSBs since DSB repair initially involves formation of γ‐H2AX after DNA damage, which then recruits 53BP1 (Siddiqui et al. 2015). Importantly, HME treatment alone of WJ‐MSCs significantly reduced both γ‐H2AX and 53BP1 levels compared to untreated cells, that is, HME reduced the baseline levels of DNA damage. There has also been one more study demonstrating that HME decreased ROS‐induced genotoxicity in human lymphocytes (Vatan, Celikler, and Yildiz 2011). Prevention of ROS‐induced DNA damage in cells is important since it may result in mutations and diseases such as carcinogenesis (Alhmoud et al. 2020). Furthermore, DNA damage in mesenchymal stem cells may result in senescence and apoptosis and decrease their stemness, differentiation, and self‐renewal abilities (Banimohamad‐Shotorbani et al. 2020).

It was remarkable that HME exhibited antioxidant activity at much lower concentration in the cell‐based assays than in the noncellular assays assessing compounds' direct free radical scavenging ability. However, it usually happens the opposite due, for instance, to bioactive compounds' difficulty in crossing the cell membrane and entering cells. This observation suggested that HME exerted antioxidant activity in WJ‐MSCs through additional molecular mechanisms. Thus, HME's effects on the NRF2 mechanism, one of the most important antioxidant defense pathways (Bellezza et al. 2018), were investigated. HME treatment of WJ‐MSCs significantly increased *NFE2L2* gene expression at the transcriptional level compared to untreated cells. This finding was confirmed by the increase in NRF2 protein levels in whole cell lysates of WJ‐MSCs treated with HME. Moreover, NRF2 was shown to be activated since HME treatment induced NRF2's translocation to the nuclei of WJ‐MSCs. This translocation was double confirmed by the higher immunofluorescence staining of the cells' nuclei for NRF2 and by the higher nuclear fraction's NRF2 protein levels in WJ‐MSCs treated with HME, compared to untreated cells. The present study is the first to demonstrate that HME induces the NRF2 pathway in human cells.

For further confirmation of the HME treatment‐induced NRF2 activation, the expression of eight of its target genes (i.e., *GCLC*, *GSR*, *HMOX1*, *SOD1*, *TXN*, *CAT*, *GPX1*, and *NQO1*) was examined. HME treatment significantly increased both the transcriptional and translational expressions of *GCLC*, *GSR*, *HMOX1*, *SOD1*, and *TXN* genes. Moreover, although HME treatment did not induce the mRNA levels of *GPX1* gene, it increased the levels of GPX1 enzyme. Discrepancies between mRNA and protein levels of a gene's expression are not unusual and may be due to posttranscriptional and/or (post)‐translational regulatory processes (Csárdi et al. 2015). However, since genes' function is mediated through their encoded proteins, the levels of the latter are physiologically more relevant than mRNA levels. Our results regarding increase of SOD1 and GPX1 by HME treatment were in agreement with one previous study reporting that HME administration increased SOD and GPX activities in rat tissues (Balamurugan et al. 2017).

Contrary to the above results, HME treatment did not induce the expression at the protein level of two of NRF2's target genes, namely, *CAT* and *NQO1*. The observed inability of NRF2 to induce the expression of CAT and NQO1 may be due to the fact that NRF2 does not always induce all of its target genes in every cellular context (Malhotra et al. 2010; He, Ru, and Wen 2020). Specifically, transcriptional regulation by NRF2 depends on a complex regulatory network consisting of the specific cellular environment, the presence of co‐regulators and other transcription factors, the availability of binding sites on target gene promoters, and the simultaneous activation of other signaling pathways (Malhotra et al. 2010; He, Ru, and Wen 2020).

The upregulation of the above antioxidant proteins (i.e., GCLC, GSR, HMOX1, SOD1, TXN, and GPX1) supported ΗΜΕ's protection from oxidative stress in WJ‐MSCs. Specifically, GCLC is the most important enzyme in GSH synthesis (Cassier‐Chauvat et al. 2023). Τhe enhanced GSH levels were also in accordance with the increased expression of GPX1 which uses GSH to reduce H_2_O_2_ to water and oxidized glutathione (GSSG), thus preventing ^**·**^OH formation (Handy and Loscalzo 2022). The increase in GPX1 also explained decrease in TBARS since GPX1 catalyzes the conversion of lipid hydroperoxides to water and consequently impedes lipid peroxidation (Handy and Loscalzo 2022). Moreover, the possible increase of GSSG generation due to increased GPX1 was in agreement with GSR's upregulation, which uses GSSG for GSH regeneration (Cassier‐Chauvat et al. 2023). TXN can directly scavenge free radicals and reduce disulfide bonds in proteins, consequently preventing their oxidation (Oberacker et al. 2023). TXN has also been reported to play role in DNA repair through p53 regulation, and so the increase of TXN may account, in part, for HME's protection from DNA damage in WJ‐MSCs (Kamal et al. 2016). The HMOX1 enzyme also has important antioxidant activity in cells since its catalysis of heme results in the production of the antioxidant molecules biliverdin and bilirubin (Alonso‐Piñeiro et al. 2021). Moreover, HMOX1 stimulates the expression of ferritin which stores iron and thus prevents Fe^2+^ formation, which could lead to free radical generation through the Fenton reaction (Alonso‐Piñeiro et al. 2021). SOD1, an important antioxidant enzyme, catalyzes the dismutation of O_2_^**·** *−*^ to water and H_2_O_2_, thus preventing attack of O_2_^**·** *−*^ on critical biomolecules (Saxena et al. 2022).

## 5 Conclusions

**FIGURE 8:**
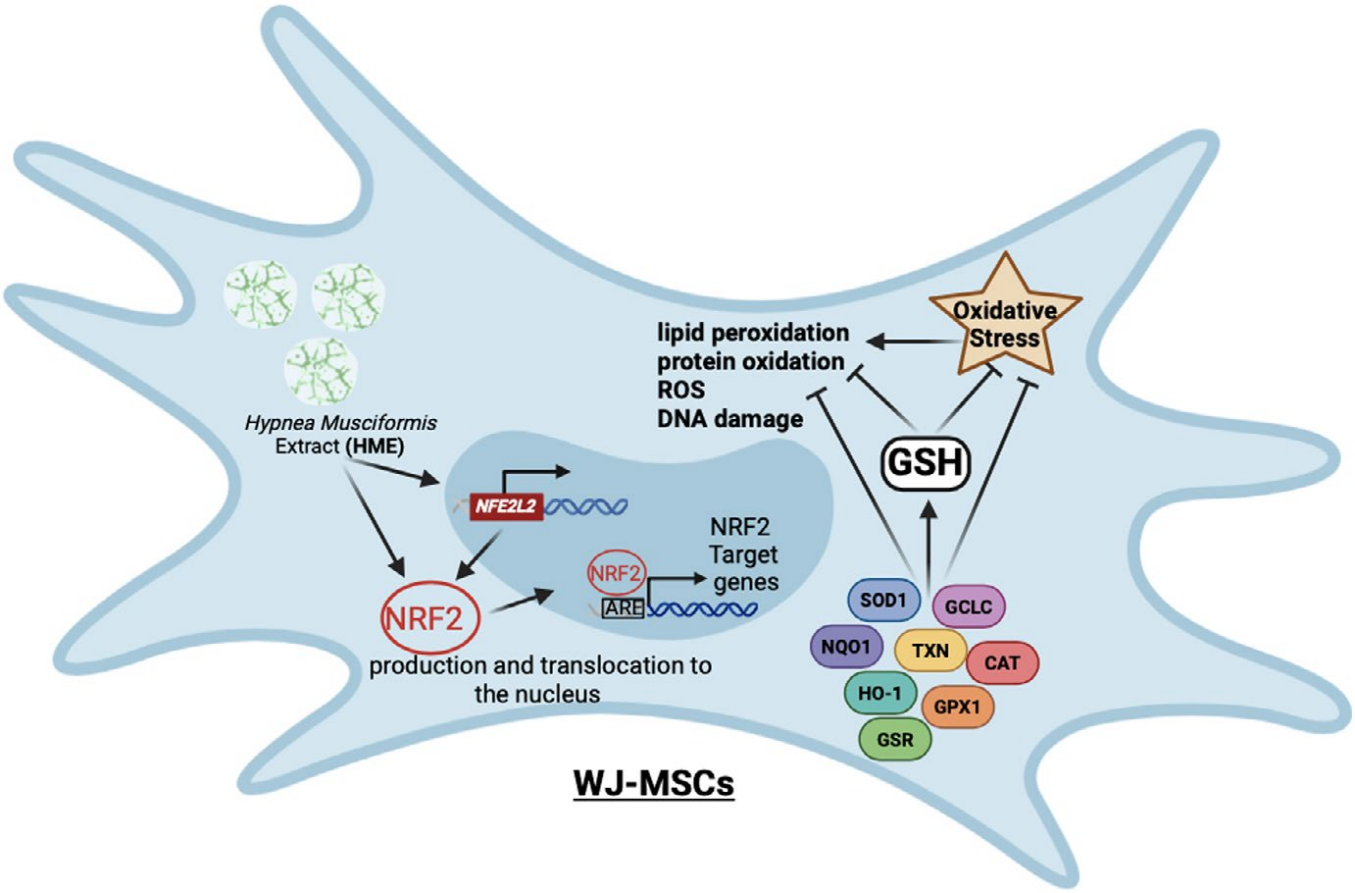
The molecular mechanism suggested by the results for HME's antioxidant activity in WJ‐MSCs.

**FIGURE A1:**
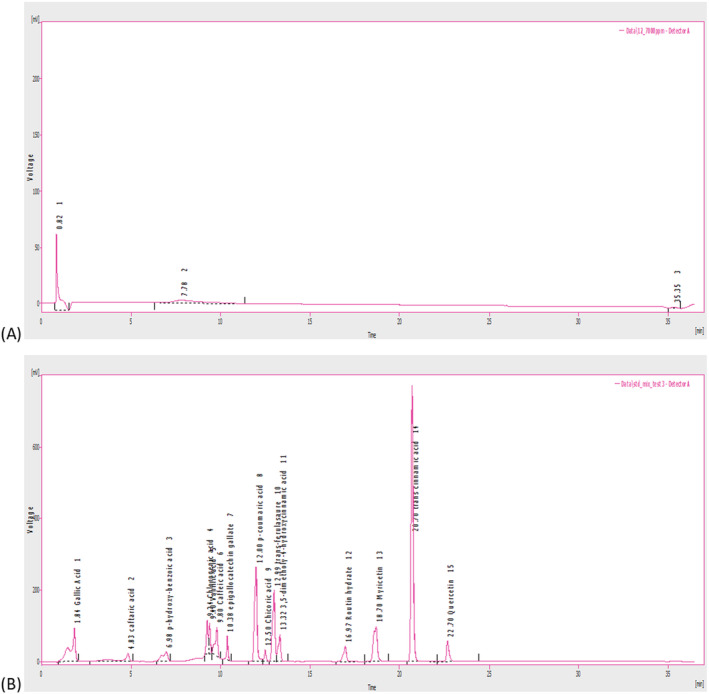
(A) HPLC chromatogram of HME at 280 nm. None of the examined polyphenols were detected. The concentration of HME sample used for the analysis was 7000 ppm (i.e., 7 mg/mL). (B) HPLC chromatogram of mixture of standards used to identify and quantify possible polyphenols present in HME. The standards included gallic acid, caftaric acid, p‐hydroxyl‐benzoic acid, chlorogenic acid, vanillic acid, caffeic acid, epigallocatechin gallate, p‐coumaric acid, chicoric acid, trans‐ferulic acid, sinapinic acid, rutin hydrate, myricetin, trans‐cinnamic acid, and quercetin.

In summary, the present study showed that the extract from *H. musciformis* collected from the Mediterranean Sea (Northern Aegean Sea, Greece) contained polyphenols, flavonoids, proteins, amino acids, organic acids, organic amides, sugar alcohols, saturated fatty acids, fatty acid esters, hydrogenated diterpene alcohols, and other organic compounds. The HME exhibited in vitro free radical scavenging and reducing abilities, as well as inhibition of ROO^**·**^‐induced DNA strand breakage. Furthermore, HME protected WJ‐MSCs from H_2_O_2_‐induced oxidative stress by decreasing TBARS, CARB, and ROS levels while increasing GSH. In addition, HME protected WJ‐MSCs from H_2_O_2_‐induced DNA damage. Our findings demonstrated for the first time that these HME's protective activities in human cells were mediated through the activation of the NRF2 transcription factor, which subsequently upregulated the expression of its target genes encoding the antioxidant proteins GCLC, GSR, HMOX1, SOD1, TXN, and GPX1 (Figure 8). These results provide new insights for the antioxidant properties of *H. musciformis* and its possible use as a food supplement or for developing biofunctional foods to prevent from oxidative stress‐induced pathologies.

## Author Contributions

**Andreas Goutas:** formal analysis (equal), investigation (equal), validation (equal), writing – original draft (equal). **Nikolaos Goutzourelas:** formal analysis (equal), investigation (equal), validation (equal). **Alkistis Kevrekidou:** investigation (equal). **Dimitrios Phaedon Kevrekidis:** investigation (equal). **Paraskevi Malea:** conceptualization (equal), investigation (equal), methodology (equal), resources (equal), supervision (equal), validation (equal), visualization (equal), writing – original draft (equal), writing – review and editing (equal). **Christina Virgiliou:** investigation (equal), writing – review and editing (equal). **Andreana N. Assimopoulou:** methodology (equal), resources (equal), supervision (equal), validation (equal), writing – original draft (equal), writing – review and editing (equal). **Varvara Trachana:** conceptualization (equal), methodology (equal), resources (equal), supervision (equal), validation (equal), writing – review and editing (equal). **Nikolaos Kollatos:** investigation (equal). **Tafa Moustafa:** investigation (equal). **Ming Liu:** writing – review and editing (equal). **Xiukun Lin:** writing – review and editing (equal). **Dimitrios Komiotis:** investigation (equal), resources (equal), supervision (equal), writing – review and editing (equal). **Dimitrios Stagos:** conceptualization (lead), formal analysis (equal), funding acquisition (lead), investigation (equal), methodology (equal), project administration (lead), resources (equal), supervision (equal), validation (equal), visualization (equal), writing – original draft (equal).

## Consent

All authors have read and agreed to the published version of the manuscript.

## Conflicts of Interest

The authors declare no conflicts of interest.

## Supporting information


Data S1.

## Data Availability

Data will be made available on request.

## Appendix A

**TABLE A1:** The sequence of primers used for the assessment of mRNA levels of *NFE2L2*, *GCLC*, *GSR*, *GPX1*, *HMOX1*, *CAT*, *SOD1*, *TXN*, *NQO1,* and *GAPDH* genes in WJ‐MSCs by qRT‐PCR.

	Forward	Reverse
NFE2L2	5′‐ATTGCCTGTAAGTCCTGGTCA‐3′	5′‐ACTGCTCTTTGGACATCATTTCG‐3′
GCLC	5′‐AGCACCCTCGCTTCAGTACC‐3′	5′‐CAATTGCCCATTCCAAATCCCAT‐3′
GSR	5′‐CCAGCTTAGGAATAACCAGCGATGG‐3′	5′‐GTCTTTTTAACCTCCTTGACCTGGGAGAAC‐3′
GPX1	5′‐CGCTTCCAGACCATTGACATC‐3′	5′‐CGAGGTGGTATTTTCTGTAAGATCA‐3′
HMOX1	5′‐TCTACCGCTCCCGCATGAAC‐3′	5′‐GCAACTCCTCAAAGAGCTGGATGT‐3′
CAT	5′‐CCAGAAGAAAGCGGTCAAGAA‐3′	5′‐TGGATGTGGCTCCCGTAGTC‐3′
SOD1	5′‐ATGGACCAGTGAAGGTGTGGG‐3′	5′‐AGTCTCCAACATGCCTCTCTTC‐3′
TXN	5′‐AGCCTTTCTTTCATTCCCTCTC‐3′	5′‐TCACACTCTGAAGCAACATCCTG‐3′
NQO1	5′‐GGGCAAGTCCATCCCAACTG‐3′	5′‐GCAAGTCAGGGAAGCCTGGA‐3′
GAPDH	5′‐GAGTCAACGGATTTGGTCGT‐3′	5′‐GACAAGCTTCCCGTTCTCAG‐3′
