# Supplementary material for: Hypnea musciformis Seaweed Extract Protected Human Mesenchymal Stem Cells From Oxidative Stress Through NRF2 Activation
Source: Food Sci Nutr. 2024 Nov 27;12(12):10816–35. doi: 10.1002/fsn3.4615 (PMC11666820; doi:10.1002/fsn3.4615)
Supplement: Supplementary file 1 — Data S1. [file FSN3-12-10816-s001.docx]

SUPPLEMENTARY FILE A1

1. Materials and methods described in detail

1.1. Hypnea musciformis collection and extract preparation

H. musciformis was collected from June to September 2020 from the Thermaikos Gulf (40°40′64.46″ N, 22°89′34.38″ E; Thessaloniki, Greece), Northern Aegean Sea, Mediterranean Sea. The identification of the H. musciformis was based on various floral catalogs and studies (Benhissoune et al., 2003; Nauer et al., 2014; www.algaebase).

Extract preparation from the red seaweed was performed as described in one of our previous studies (Goutzourelas et al., 2023). In particular, H. musciformis samples were ground and soaked in a solution of 80% v/v methanol (1:30 dried weight sample to solvent volume). The mixture was then subjected to sonication using an UP400S Hielscher sonicator (Teltow, Germany) for 20 minutes at 20 cycles and 70% amplitude. After sonication, the solution was placed in a shaker incubator (Innova® 40, New Brunswick Scientific; St Albans, UK) at 25 °C and 150 rpm for 48 hours. Subsequently, the extract solution was filtered using a 0.45 μm Whatman filter paper. The solvent was removed through rotary evaporation (IKA, Werke RV-06-ML; Staufen, Germany) at 30 °C and 150 rpm under reduced pressure, followed by freeze drying (CoolsafeTM, Scanvac; Allerod, Denmark) for 24 hours, resulting in the HME in powder form.

The weight of the dried extract was measured to determine the percentage yield of the extraction process, using the following equation:

| Extraction yield (%) = [dry extract (g)/dry seaweed (g)] x 100 | (1) |
| --- | --- |

The extracts were kept at −20 °C until further use.

- 1. *Determination of HME’s chemical composition*
     1. *Evaluation of total polyphenolic content (TPC)*

The TPC value of HME was evaluated spectrophotometrically at 765 nm on a Perkin Elmer Lambda 25 UV/VIS spectrophotometer (Waltham, MA, USA) by using the Folin–Ciocalteu reagent as described previously (Goutzourelas et al., 2023). For this assay, HME was dissolved in double distilled H_2_O_2_ (6 mg/mL). The tested sample alone, without the Folin–Ciocalteu reagent, was used as a negative control. The TPC was determined by constructing a standard curve that correlated absorbance values with known concentrations (ranging from 50 to 1500 μg/mL) of gallic acid. The TPC was expressed as mg of gallic acid equivalents (GAE) per g of dry weight (dw) of extract. Each measurement was carried out in triplicate and repeated three times.

*1.2.2. Evaluation of total flavonoid content (TFC)*

The TFC of HME was determined by using the spectrophotometric method of AlCl_3_, as described by Petrotos et al., (2021). In particular, 1.0 mL of HME (100 mg/mL in double distilled H_2_O_2_) was added to a glass test tube to which 3 mL of methanol, 200 μL of AlCl_3_ solution (10% w/v in water), 200 μL of potassium acetate solution (1 M) and 5.6 mL of distilled water were added. The tube was then agitated by vortex and incubated for 30 min at ambient temperature to complete the chemical reaction. The absorbance of each sample was measured spectrophotometrically at 420 nm against a blank solution containing all the reagents except HME which was replaced by distilled water. Moreover, the tested sample alone, without AlCl_3_, was used as a negative control. TFC was determined by a standard curve of absorbance values in correlation with standard concentrations (12.5–200 μg/mL) of quercetin. The TFC was expressed as mg of quercetin equivalents (QE) per g of dw of extract. Each measurement was carried out in triplicate and repeated three times.

*1.2.3. Evaluation of total proteins*

For evaluating HME’s total proteins, the Bio-Rad Bradford protein assay kit I (Bio-Rad, Hercules, CA, USA) was used according to the manufacturer’s instructions. Specifically, 25 μL of HME (200 mg/mL in water) were mixed with 200 μL of Bradford reagent in a well of 96-well plate. The mixture was incubated at 37 °C for 30 min. Then, the absorbance was assessed at 450 nm on a Perkin Elmer EnSpire Model 2300 Multilabel microplate reader (Waltham, MA, USA). A negative control consisting of the tested sample alone without Bradford reagent was used. Protein concentration was evaluated using a standard curve of absorbance values correlated with standard concentrations (125–1,500 μg/mL) of bovine serum albumin (BSA). Each measurement was performed in triplicate and on three separate occasions.

1.2.4. Assessment of individual polyphenols using high-pressure liquid chromatography-diode-array detection (HPLC-DAD)

HPLC-DAD analysis was performed to identify individual polyphenols and simple phenols in HME as described previously (Goutzourelas et al., 2023). The HPLC analysis was conducted using an ECOM analytical HPLC instrument, model ECS05 (Prague, Czech Republic). The instrument consisted of a quaternary gradient pump (ECP2010H), a gradient box with a degasser (ECB2004), and a diode array detector (ECDA2800 UV-Vis PDA Detector). Chromatographic separation of the sample was achieved using a Fortis SpeedCore column (C18, 2.6 um, 100 × 4.6 mm) (Cheshire, United Kingdom). The elution system employed Millipore water acidified with 0.1% formic acid (A) and methanol (B), with a total flow rate of 1 mL/min. The elution gradient began with 90% A, which remained constant for 5 minutes. At 8.5 minutes, it transitioned to 72% A, and at 30 minutes, it further changed to 40% A; this remained constant for 3 min. After each injection, the system was allowed to equilibrate for 3 minutes under the initial conditions. The column temperature was maintained at 25 °C, and the injection volume was 10 μL. The detection of the peaks was performed at 280, 270, 328, and 318 nm. Data analysis was performed using Clarity Chromatography Software v8.2 (DataApex Ltd., Thessaloniki, Greece).

To identify and quantify phenolic compounds present in HME, a mixture of standards was utilized. The standards included caftaric acid, caffeic acid, epigallocatechine gallate, p-coumaric acid, chicoric acid, trans-ferulic acid, quercetin, sinapinic acid, rutin hydrate, trans-cinnamic acid, gallic acid, p-hydroxyl-benzoic acid, chlorogenic acid, vanillic acid and myricetin (Merck, Darmstadt, Germany). These standards were diluted in methanol and subjected to analysis at wavelengths of 280 nm, 270 nm, 328 nm, and 318 nm. Each calibration curve was constructed using a mixture of standards ranging in concentration from 0.78 ppm to 200 ppm. Phenolic content analysis of the HME was performed using a concentration of 7.0 mg/mL in methanol, and the identified compounds were compared against the standards.

1.2.5. Assessment of HME’s chemical composition using gas chromatography mass spectrometry (GC-MS) analysis

HME’s chemical constitution was evaluated using GC-MS analysis. Specifically, 1 mg of dried extract was diluted with 200 μL methanol. For aromatic profile analysis, the sample was added to a GC-MS vial. Polar content analysis was based on a two step derivatisation procedure performed before sample analysis. Specifically, an aliquot of 50 μL was added to a glass vial and evaporated to dryness using speedVac. Methoxymation was performed by the addition of 10 μL MeOX (methoxyamine, 40 mg/mL) at 40 °C for 90 min. Then, silylation was carried out after the addition of MSTF 1% TMCS, at 90 °C for 30 min. Samples were left for 30 min at room temperature before analysis. Pentadecane was used as an injection standard.

Once sample was prepared, analysis was carried out on an EVOQ 456 GC-TQ-MS system (Bruker, Billerica, MA, USA) equipped with a CTC automatic sampler and PTV injector, controlled by Compass Hystar software. An HP-INNOWAX (30 m × 0.25 mm × 0.25 μm) column (Agilent Technologies, Santa Clara, CA) was used for the aromatic characterization of the sample. For derivatised samples, a 30 m HP-5 MS UI (Agilent J&W) column (0.25 mm, ID of 0.25 μm) was used, into which 1 μL of sample was injected in splitless mode. The carrier gas was helium (99.999%), used at a flow rate of 1.1 mL/min. For the untargeted analysis, the initial inlet temperature was 110 °C for 1 min and then increased to 250 °C at a rate of 250 °C/min, where it was held for 12 min. The temperature then returned to the initial conditions for the remaining 24.6 min of the run. The column temperature was set to 60 °C for the initial 1 min before increasing to 320 °C at 10 °C/min. The column then returned to the initial temperature and was held for 1 min. The total analysis time was 38 min. Electron ionization (EI) was applied, and the ion source and transfer line temperatures were set to 230 °C and 250 °C, respectively. Mass spectra were acquired over the range of 50–600 amu in full scan mode, with a solvent delay of 6.8 min. Chromatographic data were processed using MSWS data processing software (Bruker Daltonics, Bremen, Germany) and the NIST17 Mass Spectral Library (mainlib library). In addition, the open-source software pipeline MS-DIAL (RIKEN CSRS/IMS, Tokyo, Japan) (Lai et al., 2018) was used for the identification and quantification of small molecules by mass spectral deconvolution. Chromatographic peak areas of Extracted Ion Chromatograms (EICs) together with possible identities and retention times were exported and further inspected in Microsoft Excel. The identification of most of the detected compounds was confirmed by the analysis of stable standards. For the compounds without standards, the identification probability % was calculated by the NIST17 library.

1.3. Free radical scavenging assays

The DPPH^•^ assay was performed as described previously (Goutzourelas et al., 2023). In particular, HME was dissolved in double distilled water at a concentration of 300 mg/mL. Serial dilutions of the stock solution were made to obtain different extract concentrations. Then, 100 μL of each concentration was added to methanolic solution of the DPPH^•^ radical (100 μM), resulting in a final volume of 1 mL. After vortexing the samples, they were incubated at room temperature in the dark for 20 min, and the absorbance was measured at 517 nm using a Perkin Elmer Lambda 25 UV/VIS spectrophotometer (Waltham, MA, USA). In each experiment, a negative control was included, which consisted of the tested sample alone in methanol. A control solution containing only DPPH^•^ in methanol was also used. Ascorbic acid was employed as a positive control to evaluate the antioxidant activity.

The percentage of radical scavenging capacity (RSC) of the tested samples was evaluated according to the following formula:

| RSC (%) = [(A_control_ – A_sample_)/A_control_] x 100 | (2) |
| --- | --- |

where A_control_ and A_sample_ are the absorbance values of the control and the sample, respectively. The IC_50_ value representing the concentration at which 50% of the DPPH^•^ radical scavenging occurred, was calculated from the graph plotted as RSC percentage against the extract concentration. The experiment was repeated on at least three different occasions.

The ABTS^•+^ radical scavenging assay was carried out as described previously (Goutzourelas et al., 2023). In summary, the ABTS^•+^ radical was produced by combining 2 mM ABTS, 30 μM hydrogen peroxide (H_2_O_2_), and 6 μM horseradish peroxidase (HRP) enzyme in 1 mL of distilled water. The mixture of reagents was thoroughly mixed and left to incubate at room temperature in the dark for 45 min. HME was dissolved in double distilled water at a concentration of 300 mg/mL. Serial dilutions of the stock solution were made to obtain different extract concentrations. Subsequently, 10 μL of the different extract concentrations were added to the reaction mixture, and the absorbance was measured at a wavelength of 730 nm. In each experiment, the tested sample in distilled water containing ABTS^•+^ and H_2_O_2_ was used as a negative control. The ABTS^•+^ radical solution with 10 μL of H_2_O was used as control. Ascorbic acid was used as a positive control for the free radical scavenging activity. The percentage of RSC and the IC_50_ value of the tested extract were calculated as described above for the DPPH assay. The experiment was repeated on at least three different occasions.

The ^•^OH scavenging activity was determined as described previously (Goutzourelas et al., 2023). In particular, different concentrations of HME dissolved in distilled water (75 μL) were added to a mixture consisting of 450 μL sodium phosphate buffer (0.2 M, pH 7.4), 150 μL 2-deoxyribose (10 mM), 150 μL FeSO_4_-EDTA (10 mM), 525 μL H_2_O, and 150 μL H_2_O_2_ (10 mM). The samples were then incubated at 37 °C for 4 hours. Following the incubation, 750 μL trichloroacetic acid (TCA) (2.8%) and 750 μL 2-thiobarbituric acid (1%) were added, and the samples were incubated at 95 °C for 10 min. After cooling the samples on ice for 5 min, they were centrifuged at 3,000 rpm for 10 min at 25 °C. The absorbance was measured at 520 nm. Negative controls were included in each experiment, where samples without H_2_O_2_ were used. The samples without extract were used as controls. Ascorbic acid was used as a positive control for the antioxidant activity. The RSC and the IC_50_ values for ^•^OH assay were evaluated as mentioned above for the DPPH^•^ radical. The experiment was repeated on at least three different occasions.

The O_2_^•−^ scavenging activity of the extracts was evaluated as described previously (Goutzourelas et al., 2023). In this assay, HME was dissolved in a Tris-HCl buffer (16 mM, pH 8.0) at various concentrations. The reaction mixture was consisted of 125 μL of NBT_2_^+^ (300 μΜ), 125 μL of NADH (468 μΜ), and 10 μL of extract (diluted in the buffer) added to 615 μL of Tris-HCl (16 mM; pH 8.0). The reaction was initiated by adding 125 μL of PMS (60 μΜ) to the mixture. The samples were then incubated in the dark for 5 min, and the absorbance was measured at 560 nm on a Perkin Elmer Lambda 25 UV/VIS spectrophotometer (Waltham, MA, USA). Each measurement included a blank containing 750 μL of Tri-HCl buffer, 125 μL of NBT, and 125 μL of NADH, as well as a control containing 625 μL of Tri-HCl buffer, 125 μL of NBT, 125 μL of NADH, and 125 μL of PMS. Negative controls were also included in each experiment, consisting of 740 μL of Tri-HCl buffer, 125 μL of NBT, 125 μL of NADH, and 10 μL of extract diluted in buffer. The RSC and the IC_50_ values for O_2_^•−^ were determined in the same manner as mentioned above for the DPPH^•^ radical. The experiment was repeated on at least three different occasions.

*1.4.* *Reducing power (RP) assay*

Reducing power was determined spectrophotometrically as described previously (Goutzourelas et al., 2023). In this assay, HME was dissolved in phosphate buffer (0.2 M, pH 6.6) at various concentrations. Two hundred and fifty microliters of the extract solution were combined with 250 μL of potassium ferricyanide (1% *w*/*v* in dH_2_O) and incubated at 50 °C for 20 min. After incubation, the samples were cooled on ice for 5 min. Subsequently, 250 μL of TCA (10% *w*/*v*) was added, and the samples were centrifuged (1,700 g, 10 min, 25 °C). Following centrifugation, 250 μL of distilled H_2_O and 50 μL of ferric chloride (0.1% *w*/*v*) were added to the supernatant, and the samples were incubated at room temperature (RT) for 10 min. The absorbance was measured at 700 nm on a Perkin Elmer Lambda 25 UV/VIS spectrophotometer (Waltham, MA, USA). Each measurement included a blank containing 500 μL of phosphate buffer, 250 μL of TCA, 250 μL of dH_2_O, and 50 μL of ferric chloride, as well as a control containing 250 μL of buffer, 250 μL of potassium ferricyanide, 250 μL of TCA, 250 μL of dH_2_O, and 50 μL of ferric chloride. Negative controls were also included in each experiment, consisting of 250 μL of buffer, 250 μL of TCA, 250 μL of dH_2_O, 50 μL of ferric chloride and 250 μL of extract diluted in buffer. The RP_0.5AU_ value, indicating the extract concentration caused an absorbance of 0.5 at 700 nm, was determined from the graph plotting absorbance against extract concentration. The experiment was repeated on at least three different occasions.

*1.5. ROS-induced DNA plasmid strand cleavage assay*

The ROS-induced DNA plasmid strand cleavage assay was performed as described previously (Kreatsouli et al., 2019). Specifically, peroxyl radicals (ROO^•^) were generated by thermally decomposing 2,2′-azobis(2-amidinopropane hydrochloride) (AAPH). The reaction mixture (10 μL) contained 1 μg Bluescript-SK+ plasmid DNA, 2.5 mM AAPH in phosphate-buffered saline (PBS) and HME at various concentrations. The mixture was then incubated in the dark for 45 min at 37 °C. To halt the reaction, 3 μL of loading buffer (containing 0.25% bromophenol blue and 30% glycerol) was added. The DNA samples were subsequently subjected to agarose gel electrophoresis, photographed, and analyzed using the ImageJ software (National Institutes of Health, Bethesda, MD, USA). Additionally, the extract, at the highest concentration used in the assay, was applied alone without AAPH to plasmid DNA to assess its impact on plasmid DNA conformation. The percentage of the inhibitory activity of HME from ROO^•^-induced DNA strand breakage was evaluated using the following equation:

| % Inhibition = [(S − S_o_)/(S_control_ − S_o_)] × 100 | (4) |
| --- | --- |

where, S_control_ is the percentage of supercoiled DNA in the negative control (plasmid DNA alone), S_o_ is the percentage of supercoiled plasmid DNA in the positive control (without the extract but in the presence of the radical initiating factor), and S is the percentage of supercoiled plasmid DNA in the tested extract along with the radical initiating factor. Furthermore, the IC_50_ values representing the concentration that resulted in a 50% inhibition of AAPH-induced DNA relaxation, were determined. The experiment was repeated on at least three different occasions.

1.6. Primary cultures of mesenchymal stem cells

Mesenchymal stem cells (WJ-MSCs) were obtained from the Wharton Jelly of umbilical cords from term-gestation newborns after birth, having obtained consent from the parents (three different individuals, n = 3) in accordance with the principles of the Declaration of Helsinki as previously described (Tsagias et al., 2011). Isolated WJ-MSCs were cultured, as reported previously (Goutas et al., 2023), in Dulbecco’s modified Eagle’s medium DMEM high glucose with stable glutamine and sodium pyruvate (BioWest, Miami, FL, USA) plus 10% fetal bovine serum (FBS; Thermo Fisher Scientific, Waltham, MA, USA) and 1% penicillin-streptomycin (Thermo Fisher Scientific, Waltham, MA, USA) at 37 °C in a humidified atmosphere of 5% CO_2_. Middle passage WJ-MSCs (15 < p < 40) were used for the experiments. The medium was changed twice a week and cells were passaged when confluency was reached.

1.7. XTT Assay for inhibition of cell viability

The inhibition of cell proliferation was assessed using the XTT assay kit (Roche, Germany), as described previously (Goutzourelas et al., 2023). In particular, 1 × 10^4^ cells were seeded into a 96-well plate in DMEM medium. After 24 h of incubation, the cells were exposed to various concentrations of HME in FBS-free DMEM medium for 24 h. Then, a mixture consisting of 50 μL of XTT-labeling reagent and 1 μL of electron coupling reagent was added to each well, followed by 4 h incubation. The absorbance was measured at 450 nm, with a reference wavelength of 690 nm, using a Perkin Elmer EnSpire Model 2300 Multilabel microplate reader (Waltham, MA, USA). Negative controls were cells cultured only in DMEM serum-free medium without the extract. Additionally, the absorbance of the extract alone in serum-free DMEM medium and XTT test solution was measured at 450 nm. The absorbance values obtained from the extract alone were subtracted from the absorbance values of the cells treated with the extract. The data were expressed as a percentage of inhibition using the following formula:

| Inhibition (%) = [(O.D._control_ – O.D._sample_)/O.D._control_] x 100 | (5) |
| --- | --- |

where O.D._control_ and O.D._sample_ indicated the optical density of the negative control and the tested extract, respectively. The experiment was performed in triplicate and repeated on at least three different occasions.

*1.8. Cell treatment with HME for assessing effects on redox status, DNA damage, gene and protein expression*

To evaluate HME’s antioxidant activity in WJ-MSCs’, cells were seeded into 75 cm^2^ flasks containing culture medium and incubated for 24 h at 37 °C in 5% CO_2_. Different concentrations of HME dissolved in culture medium were then incubated with WJ-MSCs for 24 h. Following this, WJ-MSCs were collected using trypsin and used to assess lipid peroxidation, total antioxidant capacity (TAC), GSH levels and protein oxidation. For assessing HME’s ability to protect from oxidative stress, after 24 h treatment of WJ-MSCs with HME, the culture medium was removed and 400 μΜ of H_2_O_2_ in DMEM without FBS was added to the cell culture for 30 min.

Total ROS levels and DNA damage were also assessed in WJ-MSCs. In these cases, although the cells were incubated with HME at 100 μg/mL and/or H_2_O_2_ as described above, they were cultured in 6-well plates (2 x 10^5^ cells per well).

The expression of antioxidant genes and proteins was also assessed in WJ-MSCs. In these cases, the cells were treated with HME at 100 μg/mL and seeded into 25 cm^2^ flasks in culture medium.

1.9. Thiobarbituric acid reactive substances (TBARS), protein carbonyls (CARB), GSH and TAC assays

After treatment with HME and/or H_2_O_2_, the cells were detached using trypsin, mixed with a PBS buffer and disrupted by vigorous vortexing. The protein concentration in the resulting cell lysates was determined using the Bradford assay. Next, a slightly modified TBARS assay as described previously (Kolonas et al., 2023) was employed. Specifically, for the assay, a total of (400-X) µl of PBS (where X represents the quantity of cell suspension required to obtain 30 µg of protein) or 400 µL of PBS for the blank was combined with 500 µl of Tris-HCl (200 mM, pH 7.4) and 500 µL of 35% TCA. The mixture was then incubated for 10 min at room temperature. Subsequently, a 1 mL solution consisting of 2 M Na_2_SO_4_ and 55 mM thiobarbituric acid was added and the samples were incubated at 95 °C for 45 min. After cooling the samples on ice for 5 min and vortexing, 1 mL of 70% TCA was added. The samples were then subjected to centrifugation at 15,000 x g for 3 min, and the absorbance of the resulting supernatant was measured at 530 nm. The concentration of TBARS was calculated based on the molar extinction coefficient of malondialdehyde and expressed as TBARS nmol per mg of protein in the cell lysate. Each experiment was repeated at least three times.

For the assessment of protein oxidation, CARB levels were assessed as described previously (Kerasioti et al., 2016). In brief, following the trypsinization process, the cellular suspension was homogenized by subjecting it to sonication while kept on ice. Then, 200 µL of 20% TCA was added to 200 µL of the cellular suspension, and this mixture was incubated in an ice bath for 15 min. Afterwards, it was centrifuged at 15,000 g for 5 min at 4 °C. The resulting supernatant was discarded, and 500 µL of 2,4-Dinitrophenylhydrazine (DNPH) [in 2.5 N hydrochloride (HCl)] was added to the pellet for the sample, while 500 µL of 2.5 N HCl was added for the blank. The samples were then incubated in the dark for 1 hour, with intermittent vortexing every 15 min, and subsequently centrifuged at 15,000 g for 5 min at 4 °C. The supernatant was once again discarded, and 1 mL of 10% TCA was added to the samples, which were vortexed and centrifuged at 15,000 g for 5 min at 4 °C. After discarding the supernatant, 1 mL of ethanol-ethyl acetate (1:1 v/v) was added to the samples, which were vortexed and centrifuged at 15,000 g for 5 min at 4 °C. This washing step was repeated twice. The supernatant was discarded, and 1 mL of 5 M urea (pH 2.3) was added to the samples, which were then vortexed and incubated at 37 °C for 15 min. Subsequently, the samples were centrifuged at 15,000 g for 3 min at 4 °C, and the absorbance was measured at 375 nm. It should be noted that the assay necessitates a minimum of 30 µg of protein in the tested sample. The total protein content in the cellular suspension was determined using the Bradford assay. The calculation of CARB concentration was based on the molar extinction coefficient of DNPH. Each experiment was repeated at least three times.

GSH levels were determined as described previously (Kolonas et al., 2023). Following the trypsinization process, the cells were suspended in a PBS buffer and then lysed using sonication. Subsequently, the protein concentration in the cell lysates was determined using the Bradford assay. The next step involved performing the GSH assay, wherein a reaction mixture of 1 mL was prepared. This mixture consisted of 520 μL of 67 mM sodium phosphate buffer (pH = 8.0), 150 μl of cell lysate suspension containing 30 μg of protein, and 330 μL of 1 mM 5,5'-dithiobis(2-nitrobenzoic acid) (DTNB) solution. The samples were thoroughly mixed and incubated at room temperature in the dark for 15 min, while the absorbance was monitored at 412 nm. The concentration of GSH was then calculated based on the millimolar extinction coefficient of DTNB and expressed as nmol GSH per mg of protein in the cell lysate. Each experiment was repeated at least three times.

TAC levels were determined as described previously (Kolonas et al., 2023). After the trypsinization process, the cells were suspended in a PBS buffer and then lysed using sonication. The protein concentration in the cell lysates was determined using the Bradford assay. Subsequently, the TAC assay's reaction was carried out in a 1 mL volume, which included 50 μL of cell lysate containing 30 μg of protein, 450 μL of 10 mM sodium phosphate buffer (pH = 7.4), and 500 μL of 0.1 mM DPPH^•^ radical solution. The control samples consisted only of the radical solution that were diluted in the sodium phosphate buffer (pH = 7.4). The samples were vigorously mixed and incubated in the dark at room temperature for 60 min. Afterward, they were subjected to centrifugation (20,000 g, 3 min, 4 °C), and the absorbance was measured at 517 nm. TAC was quantified as the amount of μmol of DPPH^•^ reduced to 1,1-diphenyl-2-picryldrazine (DPPH-H) by the antioxidant components present in the cell lysate, per mg of sample protein. Each experiment was repeated at least three times.

1.10. Evaluation of ROS levels using immunofluorescence microscopy

ROS levels were determined in WJ-MSCs using immunofluorescence microscopy as described previously with slight modifications (Goutas et al., 2020). In particular, after WJ-MSCs’ treatment with HME at 100 μg/mL and/or H_2_O_2_, cells were fixed in ice cold absolute methanol for 10 min at -20 °C. Then, oxidative stress was detected by staining with green fluorescent dyes from the ROS-ID Total ROS/Superoxide Detection Kit (ENZ-51010, Enzo, Farmingdale, NY, USA) according to manufacturer’s instructions. The intensity of the green dye (total ROS detection reagent) represents the level of oxidative stress. Images were taken using a ZEISS Axio Imager Z2 fluorescent microscope and were analyzed with ImageJ software. At least five randomly selected fields and at least 200 cells were analyzed by two independent observers who were blinded to the origin of the sample (untreated or treated) for each time point or any other condition. The means of their counts were used for the statistical analysis.

1.11. RNA extraction and quantitative real-time PCR (qRT-PCR)

Total cellular RNA was purified from cultured WJ-MSCs using Trizol reagent (Invitrogen, Life Technologies, Paisley, UK) according to the manufacturer’s instructions. All samples used in the study were prominently featured with 28S and18S rRNA components. After the spectrophotometrically quantification of the yield, transcription of 1 μg RNA to complementary DNA (cDNA) was performed using SuperScript III reverse transcriptase (Invitrogen, Life Technologies, Paisley, UK) and random primers (Invitrogen, Life Technologies, Paisley, UK). Quantification of the mRNA expression of NFE2L2, SOD1, NAD(P)H quinone dehydrogenase 1 (NQO1), glutamate-cysteine ligase catalytic subunit (GCLC), GPX1, thioredoxin (TXN), glutathione-disulfide reductase (GSR), heme oxygenase 1 (HMOX1) and CAT genes was performed by real-time PCR (ABI 7300, Applied Biosystems, Fostur, Ca) using the SYBRTM Select Master Mix (Thermo Fisher Scientific Inc, Rockford, USA) according to the manufacturer’s instructions. Each analysis was performed in triplicates using 3 μL of cDNA per reaction. The oligonucleotide primers used for amplification are listed in Table A1 and were designed using Primer3 input. Calculations were performed based on the Ct method. Ct values were normalized against the endogenous reference (i.e. *GAPDH*) (ΔCt = Ct target– Ct GAPDH) and relative expression (or relative quantification RQ) was calculated using the formula RQ= 2^[-ΔΔCt], as previously described (Goutas et al., 2020).

1.12. Protein extraction and Western blot analysis

Total proteins from cells were extracted by lysing on ice with RIPA lysis buffer [10 mM Tris (pH 7.5), 150 mM NaCl, 1% Triton X-100, 1% sodium deoxycholate, 0.1% SDS, 1 mM EDTA] supplemented with a protease and phosphatase inhibitors cocktail (Thermo Fisher Scientific, Waltham, MA, USA) for 30 min. Cell lysates were then centrifuged at 12,000 rpm for 15 min at 4 °C, and supernatants were collected. Protein concentrations were quantified using bovine serum albumin (BSA) as the standard and the PierceTM BCA Protein Assay kit (Thermo Fisher Scientific, Waltham, MA, USA). A total of 20 μg protein was analysed in 10%, 12% or 14% sodium dodecyl sulfate–polyacrylamide gel electrophoresis gels (SDS–PAGE) and then transferred to polyvinylidene fluoride (PVDF) membranes (Thermo Fisher Scientific, Waltham, MA, USA). The membranes were blocked with 5% w/v nonfat dry milk in TBS/0.1% Tween20 or 5% BSA in PBS/0.1% Tween20 for 1 h at 4 °C, and then incubated overnight at 4 °C with specific primary antibodies against NRF2 (1:750 dilution, Cell Signaling Technology, #12721), SOD1 (1:1000 dilution, Cell Signaling Technology, #4266), NQO1 (1:1000 dilution, Cell Signaling Technology, #3187), GCLC (1:1000 dilution, Cell Signaling Technology, #48005), GPX1 (1:1000 dilution, Cell Signaling Technology, #3286), TXN (1:750 dilution, Cell Signaling Technology, #2429), HMOX1 (1:1000 dilution, Cell Signaling Technology, #5853), CAT (1:1000 dilution, Cell Signaling Technology, #12980), GSR (1:500 dilution, Santa Cruz Biotechnology, #sc-133245). Equal protein loading was verified by reprobing each membrane with an antibody against β-actin (1:1000 dilution, Cell Signaling Technology), which served as a loading control. In subcellular fractionation analyses, antibodies against alpha-Tubulin (1:1000 dilution, Cell Signaling Technology, #2125) and Histone H3 (1:1000 dilution, Abcam ab1791) were used as loading control for cytoplasmic and nuclear fractions, respectively. Subsequently, membranes were washed three times with TBS/0.1% Tween20 for 10 min and the appropriate secondary antibody conjugated with horseradish peroxidase [antirabbit; 1:5000 dilution, Santa Cruz Biotechnology, #sc-133245 and antimouse; 1:5000 dilution, Santa Cruz Biotechnology, #sc-525408] was then added to the membrane for 1 h at RT. Enhanced chemiluminescence was detected using ECL substrates (Thermo Fisher Scientific, Waltham, MA, USA) and visualized by Uvitec Cambridge Chemiluminescence Imaging System. The protein expression was quantified using Image J software (1.47r, Wayne Rasband National Institutes of Health, USA). Each Western blot analysis was performed at least three times and representative blots are shown. To calculate differences in protein expression between each time point or any other condition versus the No Treatment (NT) condition, protein expression levels at NT were arbitrarily set to 1.

1.13. Immunofluorescence microscopy analysis for assessing NRF2 protein

Immunofluorescence microscopy analysis was used to evaluate NRF2 protein levels. In particular, 1 x 10^5^ cells grown on coverslips in six-well plates were fixed in ice-cold absolute methanol for 10 min at -20 °C. After blocking in PBS containing 0.2% Tween20 and 1% BSA for 10 min, coverslips were incubated with a primary antibody against NRF2 (1:100, 1:750 dilution, Cell Signaling Technology, #12721) overnight at 4 °C, followed by incubation with the appropriate fluorescent dye-conjugated secondary antibody for 1 h at RT (1:500 dilution, Alexa Fluor 594, Molecular Probes). Images were taken using a ZEISS LSM 800 confocal microscope and analyzed with ImageJ software. At least five randomly selected fields and at least 200 cells were analyzed by two independent observers blinded to the origin of the sample (untreated or treated) for each time point or any other condition. The means of their counts were used for the statistical analysis.

1.14. Subcellular fractionation for assessment of NRF2

WJ-MSCs at 65–70% confluence in 25 cm^2^ flasks were treated with HME for 6, 12 and 24 h. Cells were then collected in 1.5 mL tube. Pelleted cells were resuspended in 150 μL of ice-cold buffer A (10 mM HEPES-KOH, pH 7.6) at 4 ^o^C, containing 1.5 mM MgCl_2_, 10 mM KCl, 1 mM PMSF, 0.5 mM DTT supplemented with a protease and phosphatase inhibitors cocktail (Thermo Fisher Scientific, Waltham, MA, USA) for 30 min on ice and vortexed for 10 sec every 10 min. After centrifugation (7,000 rpm 3 min 4 °C) the supernatant was collected and stored at -20 °C (cytoplasmic protein fraction). Then, 150 μL ice-cold buffer B was added to the pellet (20 mM HEPES-KOH pH 7.6 at 4 °C, 1.5 mM MgCl_2_, 420 mM NaCl, 0.2 Mm EDTA, 1 mM PMSF, 0.5 mM DTT 25% glycerol) supplemented with protease and phosphatase inhibitors cocktail (Thermo Fisher Scientific, Waltham, MA, USA) for 30 min on ice and vortexed for 10 sec every 10 min. After centrifugation (7,000 rpm, 3 min, 4 °C) the supernatant was collected and stored at -20 °C (nuclear protein fraction). Both cytoplasmic and nuclear protein fractions were used for assessing NRF2 levels using Western blot analysis as described above. Each experiment was performed at least three times, and representative blots are shown.

1.15. Assessment of protection from DNA damage in WJ-MSCs using immunofluorescence microscopy

After the treatment of WJ-MSCs with HME at 100 μg/mL and/or H_2_O_2_, DNA double-strand breaks (DSB) were assessed using immunofluorescence microscopy, as described above for NRF2 assessment, with modifications. In particular, for DSB detection, fixed slides were incubated with specific primary antibodies against 53BP1 (1:500, clone BP13, mouse monoclonal, Millipore, MA, USA) or anti-γH2AX (1:200, 05-636, clone JBW301, mouse monoclonal, Millipore, MA, USA) and the corresponding secondary antibody (1:500 dilution, Alexa Fluor 488, Molecular Probes). Coverslips were then embedded in 10 μL of Vectashield mounting medium (Vector Laboratories, Burlingame, CA, USA) containing 4,6-diamidino-2-phenylindole (DAPI) to visualize nuclei. Images were taken using a ZEISS Axio Imager Z2 fluorescent microscope and analyzed with ImageJ software, as described above for NRF2 protein. At least five randomly selected fields and at least 200 cells were analyzed by two independent observers blinded to the origin of the sample (untreated or treated) for each time point or any other condition. The means of their counts were used for the statistical analysis.

1.16. Statistical analysis

All results were expressed as mean ± standard error (mean ± S.E.). The statistical analysis was based on one-way ANOVA followed by Dunnett’s test for multiple pair-wise comparisons. Differences were considered significant at *p* < 0.05. SPSS software (version 14.0; SPSS) was used for all statistical analyses.
